# Supplementary material for: Transcriptomic profiling and machine learning uncover gene signatures of psoriasis endotypes and disease severity
Source: Commun Med (Lond). 2026 Jan 21;6:65. doi: 10.1038/s43856-025-01325-4 (PMC12852803; doi:10.1038/s43856-025-01325-4)
Supplement: Supplementary file 2 — Supplemental Information [file 43856_2025_1325_MOESM2_ESM.pdf]

a

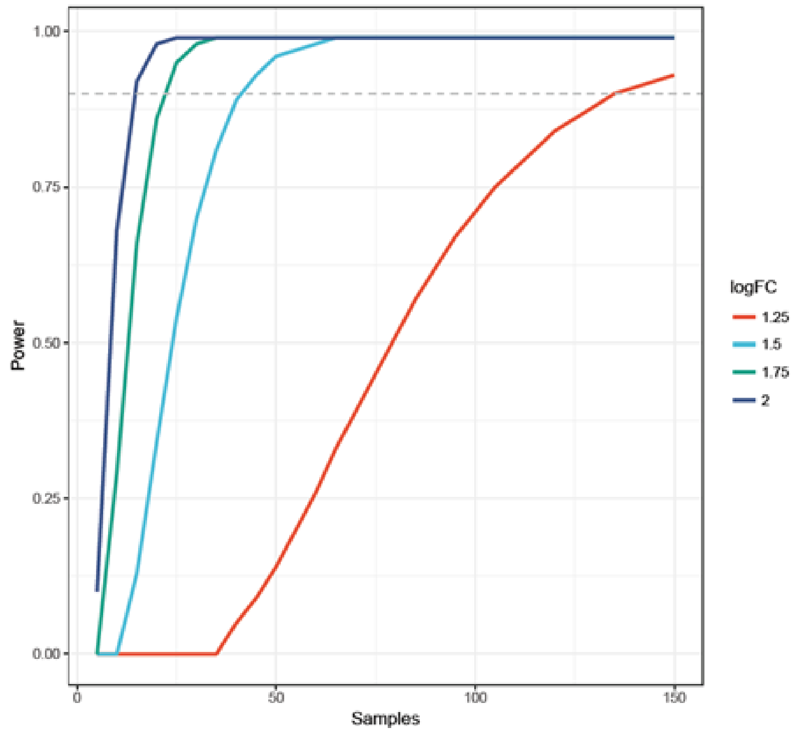

b

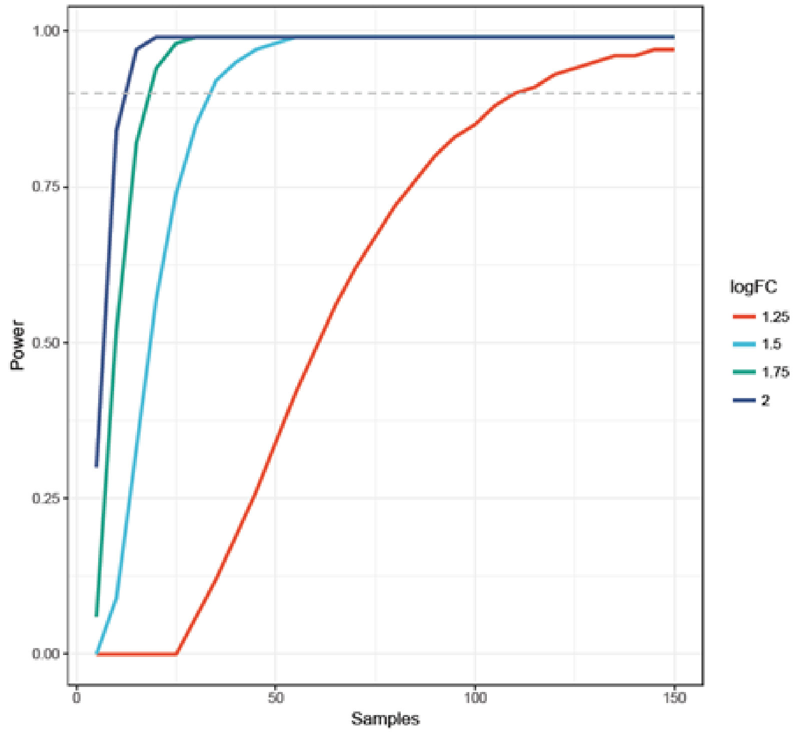

Supplementary Figure 1. Statistical power calculations for differential expression analysis.
Power curves projected across an expected range of fold changes based on the assumption that **(a)** 1% or **(b)** 5% of genes are likely to prove prognostic. Power calculations were performed using the method of Guo et al (2014) and parameters derived from our pilot study (Foulkes et al 2018); we calculated the requisite sample size to achieve 90% power (grey dotted line on plot) to detect differential expression associated with response.

b

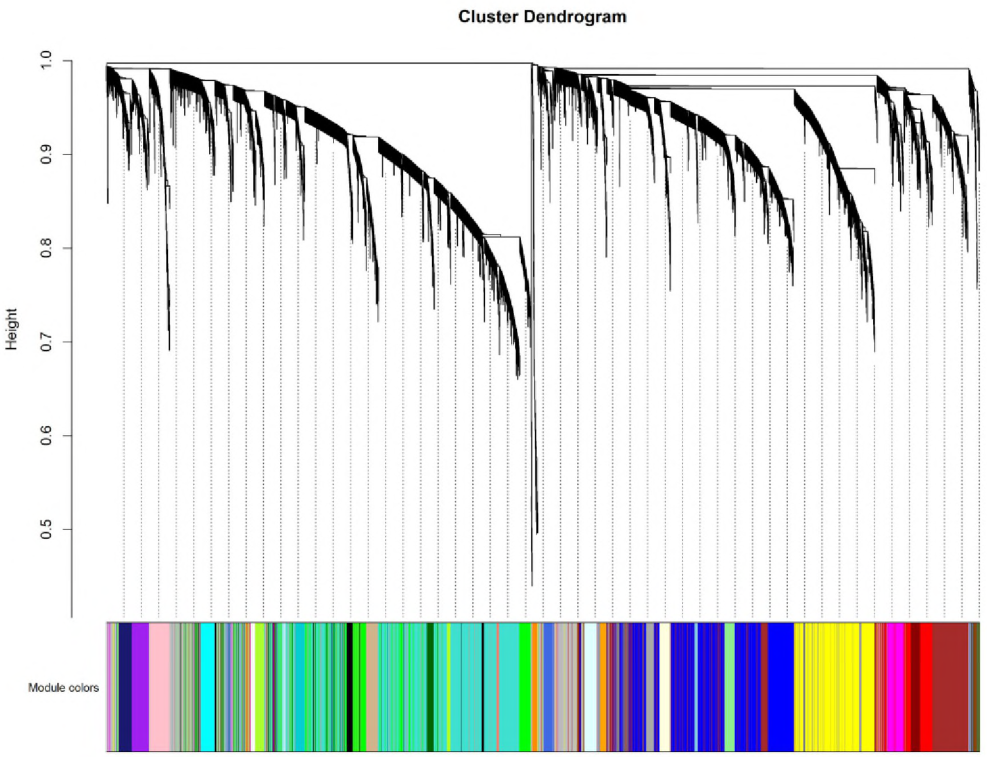
Cluster Dendrogram
Height
1.0
0.9
0.8
0.7
0.6
0.5
Module colors

b

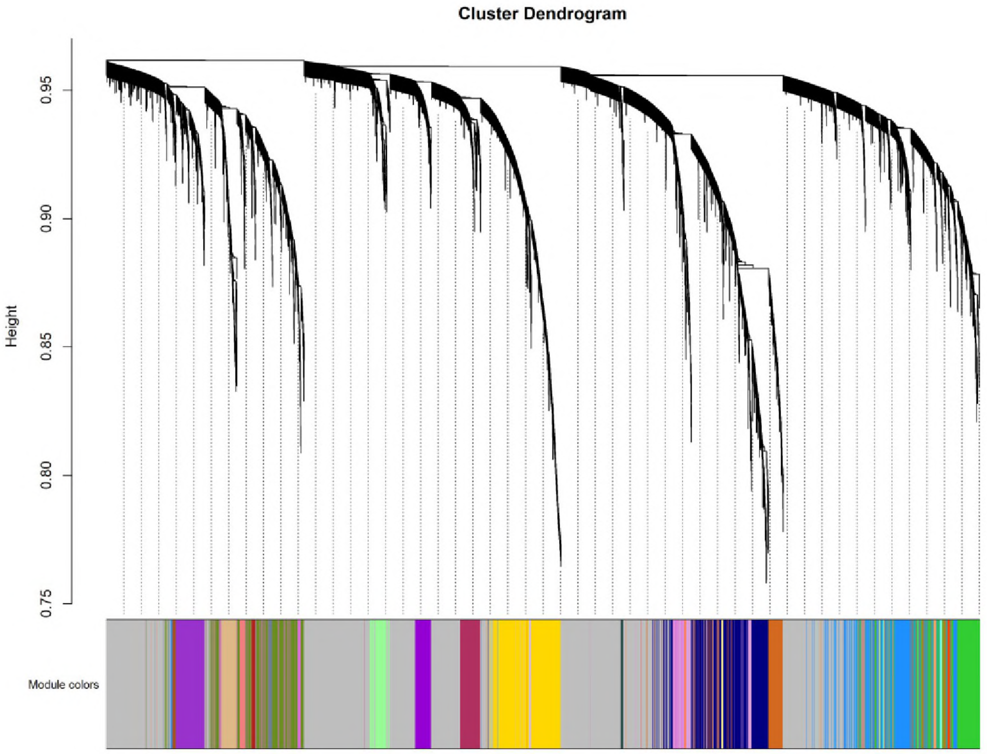
Cluster Dendrogram
Height
0.95
0.90
0.85
0.80
0.75
Module colors

Supplementary Figure 2. Grouping of genes in skin and blood into co-expressed gene modules using WGCNA.

WGCNA, a dimensionality reduction method for omics data, was used to group the skin and blood RNA-Seq data into co-expressed gene modules. Briefly, WGCNA works by calculating the correlation between each pair of genes in an expression matrix; the resulting correlation matrix, after some transformations, is then clustered, allowing genes with similar expression profiles to be grouped into modules. Further details are available in the supplementary methods. **(a)** shows hierarchical clustering of genes in the skin data, annotated by colour-coded module assignment and **(b)** shows the same for blood. WGCNA identified 34 modules in skin and 26 modules in blood.

Abbreviations: WGCNA, weighted gene correlation network analysis.

a b

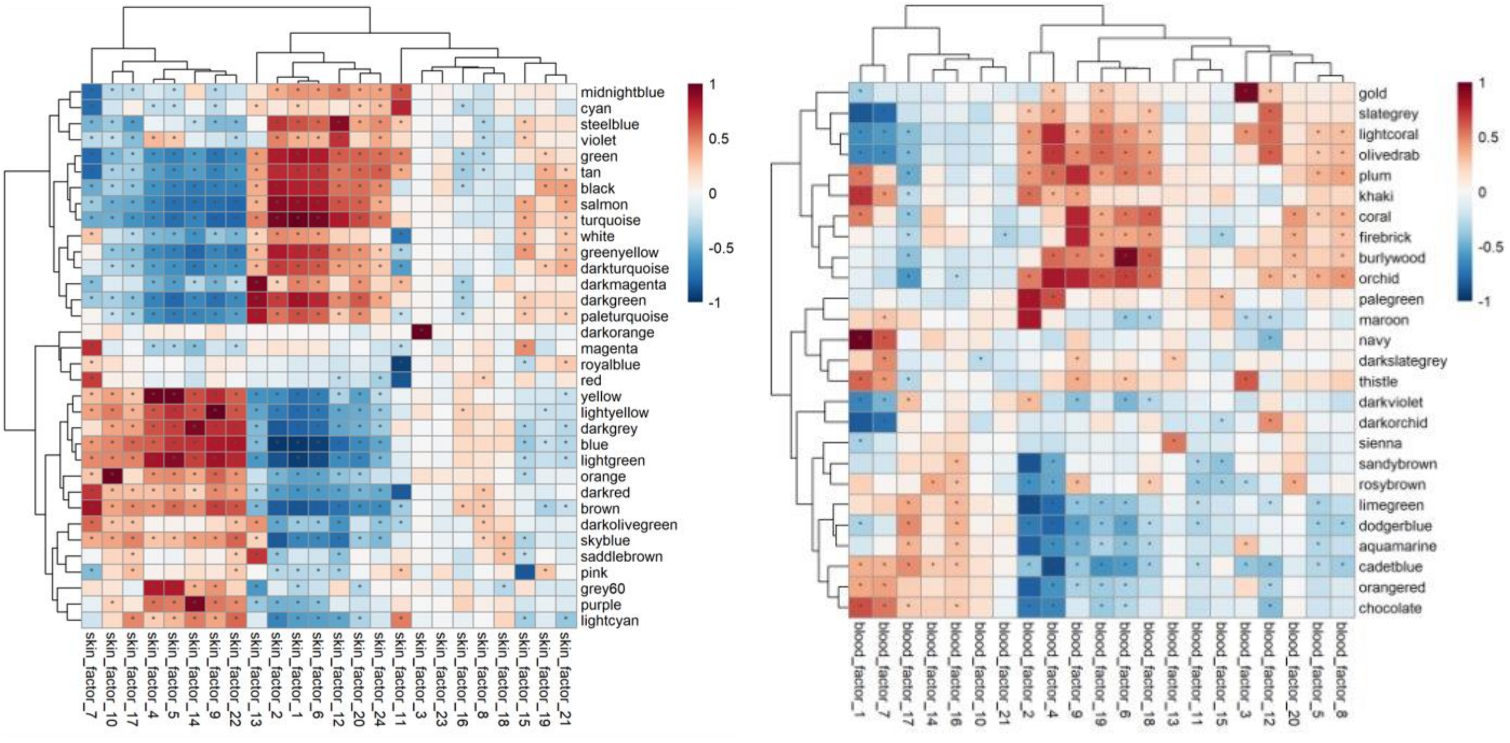

Supplementary figure 3. WGCNA and ICA identify similar gene expression signatures.
Correlations between module eigengenes (y-axis) and latent factor values (x-axis) in **(a)** skin and **(b)** blood. The high degree of positive correlation shown in these heatmaps demonstrates that WGCNA and ICA converged on similar gene expression profiles, providing cross-validation of these dimensionality reduction methods.

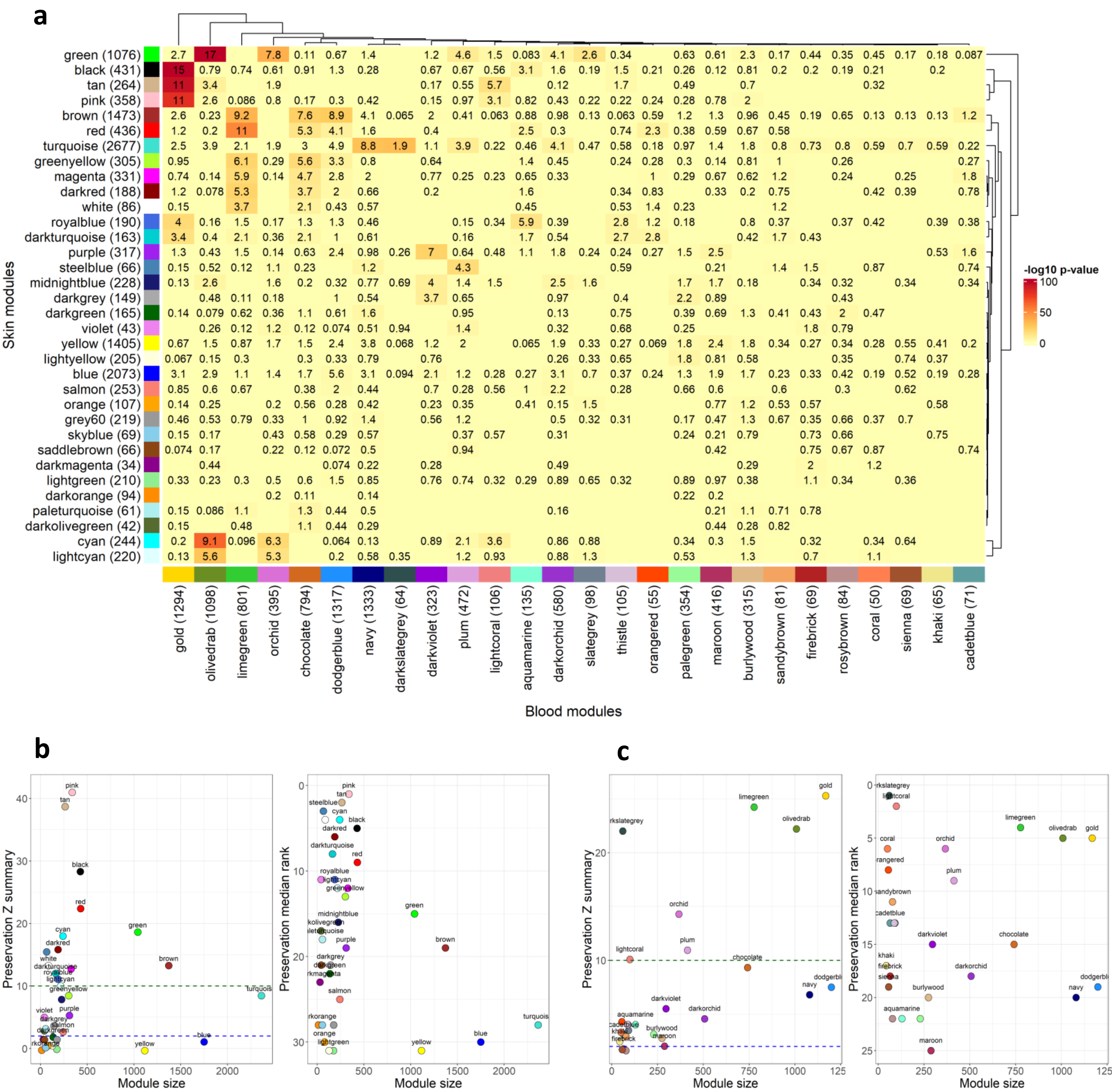
a
Skin modules
green (1076)
black (431)
tan (264)
pink (358)
brown (1473)
red (436)
turquoise (2677)
greenyellow (305)
magenta (331)
darkred (188)
white (86)
royalblue (190)
darkturquoise (163)
purple (317)
steelblue (66)
midnightblue (228)
darkgrey (149)
darkgreen (165)
violet (43)
yellow (1405)
lightyellow (205)
blue (2073)
salmon (253)
orange (107)
grey60 (219)
skyblue (69)
saddlebrown (66)
darkmagenta (34)
lightgreen (210)
darkorange (94)
paleturquoise (61)
darkolivegreen (42)
cyan (244)
lightcyan (220)
gold (1294)
olivedrab (1098)
limegreen (801)
orchid (395)
chocolate (794)
dodgerblue (1317)
navy (1333)
darkslategrey (64)
darkviolet (323)
plum (472)
lightcoral (106)
aquamarine (135)
darkorchid (580)
slategrey (98)
thistle (105)
orangered (55)
palegreen (354)
maroon (416)
burlywood (315)
sandybrown (81)
firebrick (69)
rosybrown (84)
coral (50)
sienna (69)
khaki (65)
cadetblue (71)
Blood modules
-log10 p-value
100
50
0
b
Preservation Z summary
Preservation median rank
Module size
c
Preservation Z summary
Preservation median rank
Module size

Supplementary Figure 4. Module preservation analysis identifies modules with similar patterns of co-expression in skin and blood, and vice versa.

WGCNA was used to derive co-expressed gene modules separately in skin and blood; we then sought to identify which modules in skin showed similar patterns of co-expression in blood, and vice versa. We first looked for pairs of modules between skin and blood with significant overlap in gene contents. **(a)** shows gene overlap between the skin and blood modules. The heatmap is coloured by –log10 p-values from right tailed fisher's exact test and the numbers in each cell correspond to the percentage of the union of genes in each module pair that overlap. We also used tested module preservation between the skin and blood modules using functionality available in the WGCNA R package. We derived two composite module preservation scores, preservation Z summary and median rank. **(b)** shows composite scores from testing preservation of the skin modules in blood and **(c)** shows the scores derived from testing preservation of the blood modules in skin. In each plot the left panel shows preservation Z summary and the right panel shows median rank. Each score is plotted against module size. The blue horizontal line indicates moderate evidence for preservation and the green horizontal line indicates strong evidence for preservation. The black , pink, and tan modules in skin exhibited strong evidence for preservation in blood and were found to have the most significant gene overlap with the gold module in blood. The green skin module was also strongly preserved in blood and exhibited significant gene overlap with the olivedrab module in blood.

Abbreviations: WGCNA, weighted gene correlation network analysis.

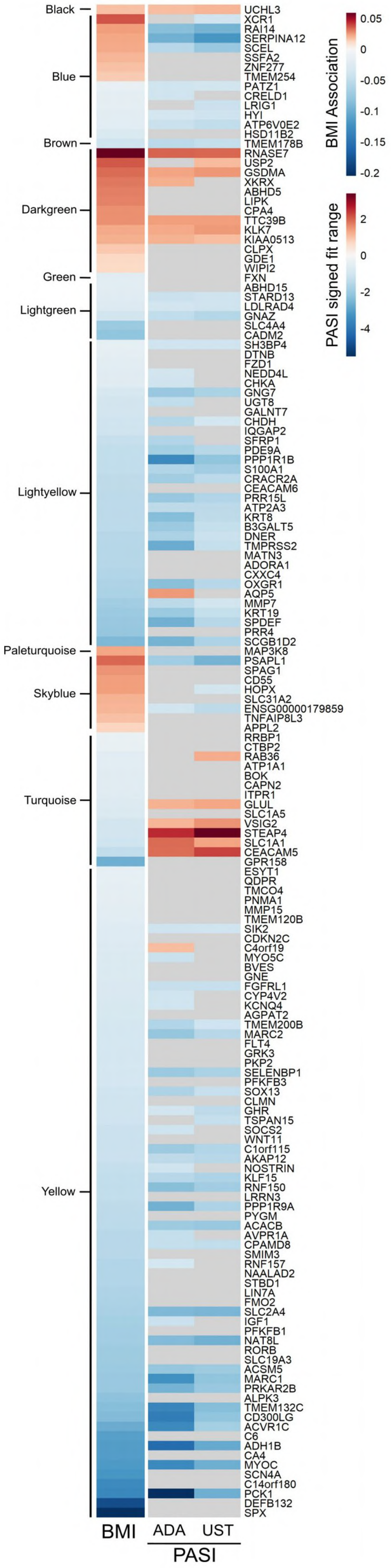
Black
Blue
Brown
Darkgreen
Green
Lightgreen
Lightyellow
Paleturquoise
Skyblue
Turquoise
Yellow
UCHL3
XCR1
RAI14
SERPINA12
SCEL
SSFA2
ZNF277
TMEM254
PATZ1
CRELD1
LRIG1
HYI
ATP6V0E2
HSD11B2
TMEM178B
RNASE7
USP2
GSDMA
XKRX
ABHD5
LIPK
CPA4
TTC39B
KLK7
KIAA0513
CLPX
GDE1
WIPI2
FXN
ABHD15
STARD13
LDLRAD4
GNAZ
SLC4A4
CADM2
SH3BP4
DTNB
FZD1
NEDD4L
CHKA
GNG7
UGT8
GALNT7
CHDH
IQGAP2
SFRP1
PDE9A
PPP1R1B
S100A1
CRACR2A
CEACAM6
PRR15L
ATP2A3
KRT8
B3GALT5
DNER
TMPRSS2
MATN3
ADORA1
CXXC4
OXGR1
AQP5
MMP7
KRT19
SPDEF
PRR4
SCGB1D2
MAP3K8
PSAPL1
SPAG1
CD55
HOPX
SLC31A2
ENSG00000179859
TNFAIP8L3
APPL2
RRBP1
CTBP2
RAB36
ATP1A1
BOK
CAPN2
ITPR1
GLUL
SLC1A5
VSIG2
STEAP4
SLC1A1
CEACAM5
GPR158
ESYT1
QDPR
TMCO4
PNMA1
MMP15
TMEM120B
SIK2
CDKN2C
C4orf19
MYO5C
BVES
GNE
FGFRL1
CYP4V2
KCNQ4
AGPAT2
TMEM200B
MARC2
FLT4
GRK3
PKP2
SELENBP1
PFKFB3
SOX13
CLMN
GHR
TSPAN15
SOCS2
WNT11
C1orf115
AKAP12
NOSTRIN
KLF15
RNF150
LRRN3
PPP1R9A
PYGM
ACACB
AVPR1A
CPAMD8
SMIM3
RNF157
NAALAD2
STBD1
LIN7A
FMO2
SLC2A4
IGF1
PFKFB1
NAT8L
RORB
SLC19A3
ACSM5
MARC1
PRKAR2B
ALPK3
TMEM132C
CD300LG
ACVR1C
C6
ADH1B
CA4
MYOC
SCN4A
C14orf180
PCK1
DEFB132
SPX
BMI
ADA
UST
PASI
BMI Association
0.05
0
-0.05
-0.1
-0.15
-0.2
PASI signed fit range
2
0
-2
-4

Supplementary figure 5. A transcriptome signature associated with BMI in non-lesional skin (NLS) is associated with disease severity in lesional skin.

This heatmap shows genes that were assigned to modules by WGCNA and exhibited a significant association with BMI in NLS based on differential expression analysis; their associations with BMI in NLS and PASI in LS in each drug cohort are shown. Red indicates a positive association, blue indicates a negative association, and grey indicates a non-significant association. Of note, genes in the lightyellow module were negatively associated with BMI in NLS and negatively associated with PASI in LS in both drug cohorts.

Abbreviations: BMI, body mass index; WGCNA, weighted gene correlation network analysis; NLS, non-lesional skin; PASI, psoriasis area and severity index; LS, lesional skin.

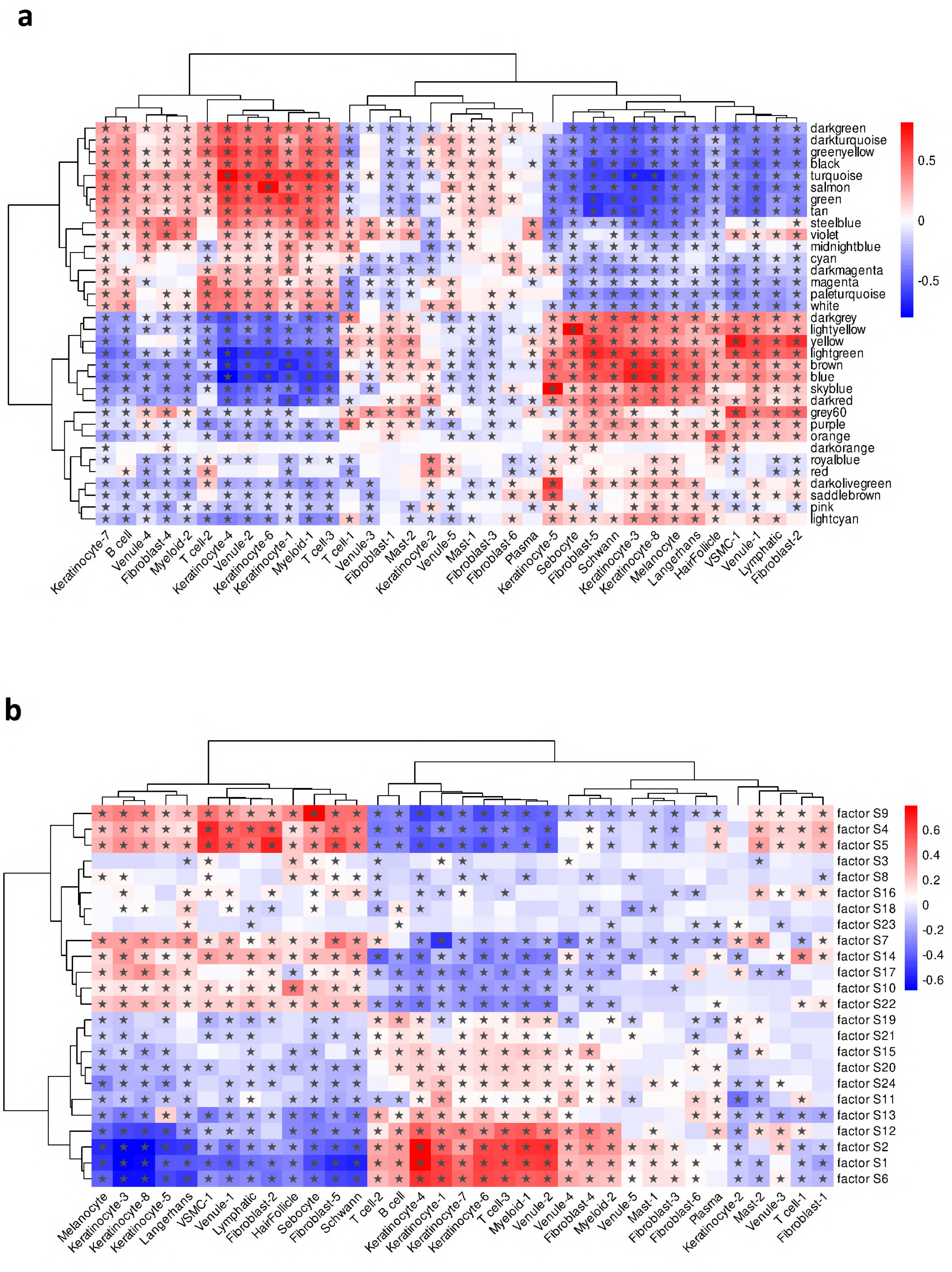
a
darkgreen
darkturquoise
greenyellow
black
turquoise
salmon
green
tan
steelblue
violet
midnightblue
cyan
darkmagenta
magenta
paleturquoise
white
darkgrey
lightyellow
yellow
lightgreen
brown
blue
skyblue
darkred
grey60
purple
orange
darkorange
royalblue
red
darkolivegreen
saddlebrown
pink
lightcyan
0.5
0
-0.5
Keratinocyte-7
B cell
Venule-4
Fibroblast-4
Myeloid-2
T cell-2
Keratinocyte-4
Venule-2
Keratinocyte-6
Keratinocyte-1
Myeloid-1
T cell-3
T cell-1
Venule-3
Fibroblast-1
Mast-2
Keratinocyte-2
Venule-5
Mast-1
Fibroblast-3
Fibroblast-6
Plasma
Keratinocyte-5
Sebocyte
Fibroblast-5
Schwann
Keratinocyte-3
Keratinocyte-8
Melanocyte
Langerhans
HairFollicle
VSMC-1
Venule-1
Lymphatic
Fibroblast-2
b
factor S9
factor S4
factor S5
factor S3
factor S8
factor S16
factor S18
factor S23
factor S7
factor S14
factor S17
factor S10
factor S22
factor S19
factor S21
factor S15
factor S20
factor S24
factor S11
factor S13
factor S12
factor S2
factor S1
factor S6
0.6
0.4
0.2
0
-0.2
-0.4
-0.6
Melanocyte
Keratinocyte-3
Keratinocyte-8
Keratinocyte-5
Langerhans
VSMC-1
Venule-1
Lymphatic
Fibroblast-2
HairFollicle
Sebocyte
Fibroblast-5
Schwann
T cell-2
B cell
Keratinocyte-4
Keratinocyte-1
Keratinocyte-7
Keratinocyte-6
T cell-3
Myeloid-1
Venule-2
Venule-4
Fibroblast-4
Myeloid-2
Venule-5
Mast-1
Fibroblast-3
Fibroblast-6
Plasma
Keratinocyte-2
Mast-2
Venule-3
T cell-1
Fibroblast-1

Supplementary Figure 6. Cell type deconvolution of the gene modules and latent factors in skin. We derived predicted cell-type fractions for the skin RNA-Seq data (discovery and replication cohorts combined) using CibersortX and a skin single cell transcriptome atlas published by Hughes et al (2020), publicly available on the Single Cell Portal (https://singlecell.broadinstitute.org/single_cell). We then correlated the predicted cell-type fractions with **(a)** module eigengenes, derived by WGCNA, and **(b)** latent factor values, derived by ICA. Asterisks (*) indicate statistical significance (adjusted p-value<0.05). *Abbreviations:* WGCNA, weighted gene correlation network analysis; ICA, independent component analysis.

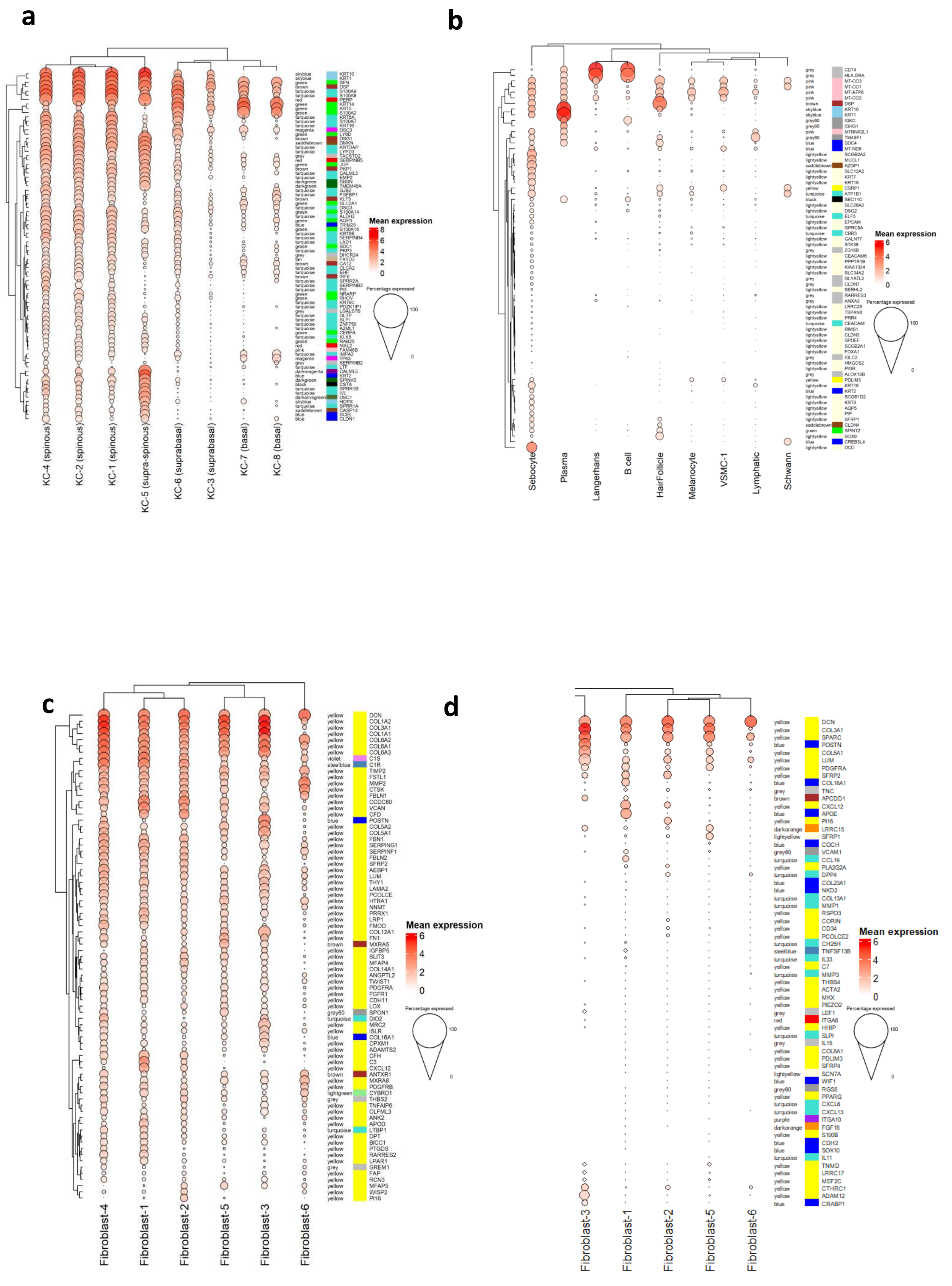
a
KC-4 (spinous)
KC-2 (spinous)
KC-1 (spinous)
KC-5 (supra-spinous)
KC-6 (suprabasal)
KC-3 (suprabasal)
KC-7 (basal)
KC-8 (basal)
Mean expression
Percentage expressed
b
Sebocyte
Plasma
Langerhans
B cell
HairFollicle
Melanocyte
VSMC-1
Lymphatic
Schwann
Mean expression
Percentage expressed
c
Fibroblast-4
Fibroblast-1
Fibroblast-2
Fibroblast-5
Fibroblast-3
Fibroblast-6
Mean expression
Percentage expressed
d
Fibroblast-3
Fibroblast-1
Fibroblast-2
Fibroblast-5
Fibroblast-6
Mean expression
Percentage expressed

e

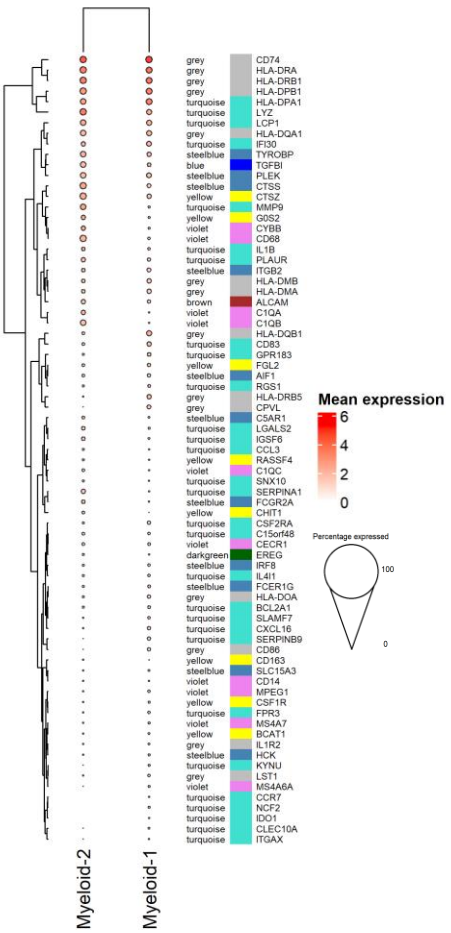
grey CD74
grey HLA-DRA
grey HLA-DRB1
grey HLA-DPB1
turquoise HLA-DPA1
turquoise LYZ
turquoise LCP1
grey HLA-DQA1
turquoise IFI30
steelblue TYROBP
blue TGFBI
steelblue PLEK
steelblue CTSS
yellow CTSZ
turquoise MMP9
yellow G0S2
violet CYBB
violet CD68
turquoise IL1B
turquoise PLAUR
steelblue ITGB2
grey HLA-DMB
grey HLA-DMA
brown ALCAM
violet C1QA
violet C1QB
grey HLA-DQB1
turquoise CD83
turquoise GPR183
yellow FGL2
steelblue AIF1
turquoise RGS1
grey HLA-DRB5
grey CPVL
steelblue C5AR1
turquoise LGALS2
turquoise IGSF6
turquoise CCL3
yellow RASSF4
violet C1QC
turquoise SNX10
turquoise SERPINA1
steelblue FCGR2A
yellow CHIT1
turquoise CSF2RA
turquoise C15orf48
violet CECR1
darkgreen EREG
steelblue IRF8
turquoise IL4I1
steelblue FCER1G
grey HLA-DOA
turquoise BCL2A1
turquoise SLAMF7
turquoise CXCL16
turquoise SERPINB9
grey CD86
yellow CD163
steelblue SLC15A3
violet CD14
violet MPEG1
yellow CSF1R
turquoise FPR3
violet MS4A7
yellow BCAT1
grey IL1R2
steelblue HCK
turquoise KYNU
grey LST1
violet MS4A6A
turquoise CCR7
turquoise NCF2
turquoise IDO1
turquoise CLEC10A
turquoise ITGAX
Mean expression
6
4
2
0
Percentage expressed
100
0
Myeloid-2
Myeloid-1

f

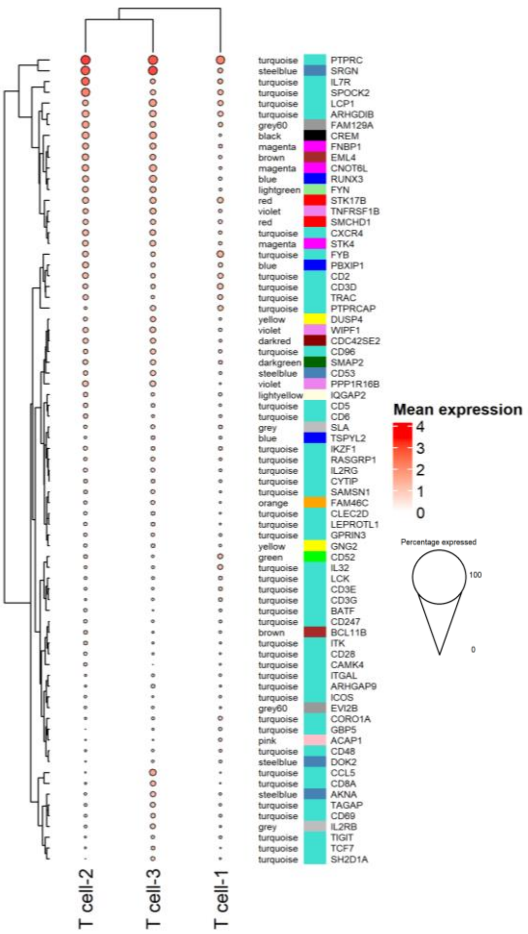
turquoise PTPRC
steelblue SRGN
turquoise IL7R
turquoise SPOCK2
turquoise LCP1
turquoise ARHGDIB
grey60 FAM129A
black CREM
magenta FNBP1
brown EML4
magenta CNOT6L
blue RUNX3
lightgreen FYN
red STK17B
violet TNFRSF1B
red SMCHD1
turquoise CXCR4
magenta STK4
turquoise FYB
blue PBXIP1
turquoise CD2
turquoise CD3D
turquoise TRAC
turquoise PTPRCAP
yellow DUSP4
violet WIPF1
darkred CDC42SE2
turquoise CD96
darkgreen SMAP2
steelblue CD53
violet PPP1R16B
lightyellow IQGAP2
turquoise CD5
turquoise CD6
grey SLA
blue TSPYL2
turquoise IKZF1
turquoise RASGRP1
turquoise IL2RG
turquoise CYTIP
turquoise SAMSN1
orange FAM46C
turquoise CLEC2D
turquoise LEPROTL1
turquoise GPRIN3
yellow GNG2
green CD52
turquoise IL32
turquoise LCK
turquoise CD3E
turquoise CD3G
turquoise BATF
turquoise CD247
brown BCL11B
turquoise ITK
turquoise CD28
turquoise CAMK4
turquoise ITGAL
turquoise ARHGAP9
turquoise ICOS
grey60 EVI2B
turquoise CORO1A
turquoise GBP5
pink ACAP1
turquoise CD48
steelblue DOK2
turquoise CCL5
turquoise CD8A
steelblue AKNA
turquoise TAGAP
turquoise CD69
grey IL2RB
turquoise TIGIT
turquoise TCF7
turquoise SH2D1A
Mean expression
4
3
2
1
0
Percentage expressed
100
0
T cell-2
T cell-3
T cell-1

g

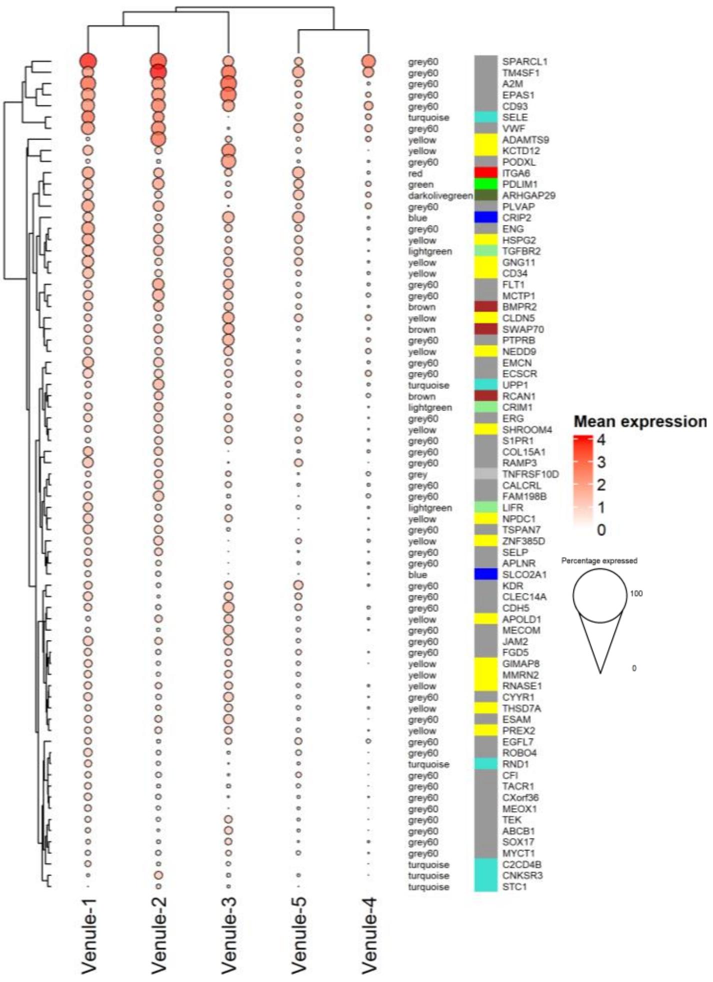
grey60 SPARCL1
grey60 TM4SF1
grey60 A2M
grey60 EPAS1
grey60 CD93
turquoise SELE
grey60 VWF
yellow ADAMTS9
yellow KCTD12
grey60 PODXL
red ITGA6
green PDLIM1
darkolivegreen ARHGAP29
grey60 PLVAP
blue CRIP2
grey60 ENG
yellow HSPG2
lightgreen TGFBR2
yellow GNG11
yellow CD34
grey60 FLT1
grey60 MCTP1
brown BMPR2
yellow CLDN5
brown SWAP70
grey60 PTPRB
yellow NEDD9
grey60 EMCN
grey60 ECSCR
turquoise UPP1
brown RCAN1
lightgreen CRIM1
grey60 ERG
yellow SHROOM4
grey60 S1PR1
grey60 COL15A1
grey60 RAMP3
grey TNFRSF10D
grey60 CALCRL
grey60 FAM198B
lightgreen LIFR
yellow NPDC1
grey60 TSPAN7
yellow ZNF385D
grey60 SELP
grey60 APLNR
blue SLCO2A1
grey60 KDR
grey60 CLEC14A
grey60 CDH5
yellow APOLD1
grey60 MECOM
grey60 JAM2
grey60 FGD5
yellow GIMAP8
yellow MMRN2
yellow RNASE1
grey60 CYYR1
yellow THSD7A
grey60 ESAM
yellow PREX2
grey60 EGFL7
grey60 ROBO4
turquoise RND1
grey60 CFI
grey60 TACR1
grey60 CXorf36
grey60 MEOX1
grey60 TEK
grey60 ABCB1
grey60 SOX17
grey60 MYCT1
turquoise C2CD4B
turquoise CNKSR3
turquoise STC1
Mean expression
4
3
2
1
0
Percentage expressed
100
0
Venule-1
Venule-2
Venule-3
Venule-5
Venule-4

Supplementary Figure 7. Expression of cell cluster marker genes in a skin single cell transcriptome atlas. Heatmaps showing the mean expression within specified cell clusters (columns) of the top 80 marker genes (ranked by average log fold change; rows) in **(a)** keratinocytes, **(b)** sebocytes, **(c)** fibroblasts, **(e)** myeloid cells, **(f)** T-cells, and **(g)** venules. This data was derived from a skin single cell transcriptome atlas published by Hughes et al (2020), publicly available on the Single Cell Portal (https://singlecell.broadinstitute.org/single_cell). The row annotation indicates the modules to which these genes were assigned in our dataset. **(d)** Shows the expression in this single cell atlas data of marker genes, identified by Steele et al (2024), which differentiate between different sub-types of fibroblasts.

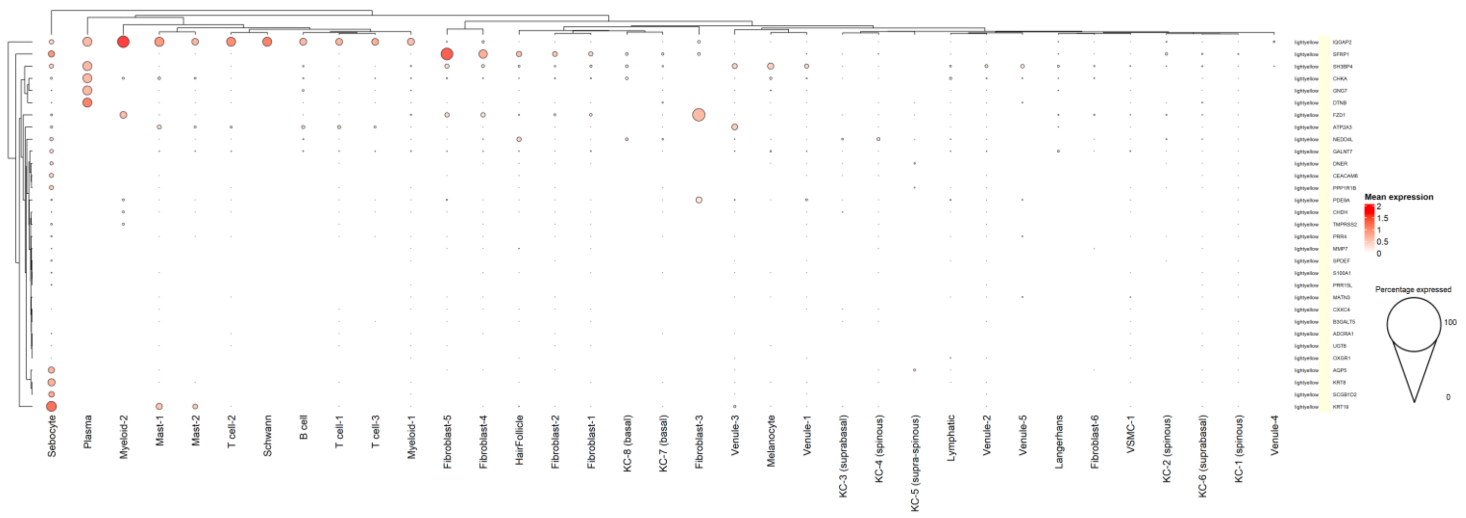

Supplementary Figure 8. Expression of BMI-associated genes in a skin single cell transcriptome atlas. Heatmaps showing the mean expression within skin cell clusters (columns) of BMI-associated DEGs that overlap with the lightyellow module. This data was derived from the skin single cell transcriptome atlas published by Hughes et al (2020), publicly available on the Single Cell Portal (https://singlecell.broadinstitute.org/single_cell).
Abbreviations: BMI, body mass index.

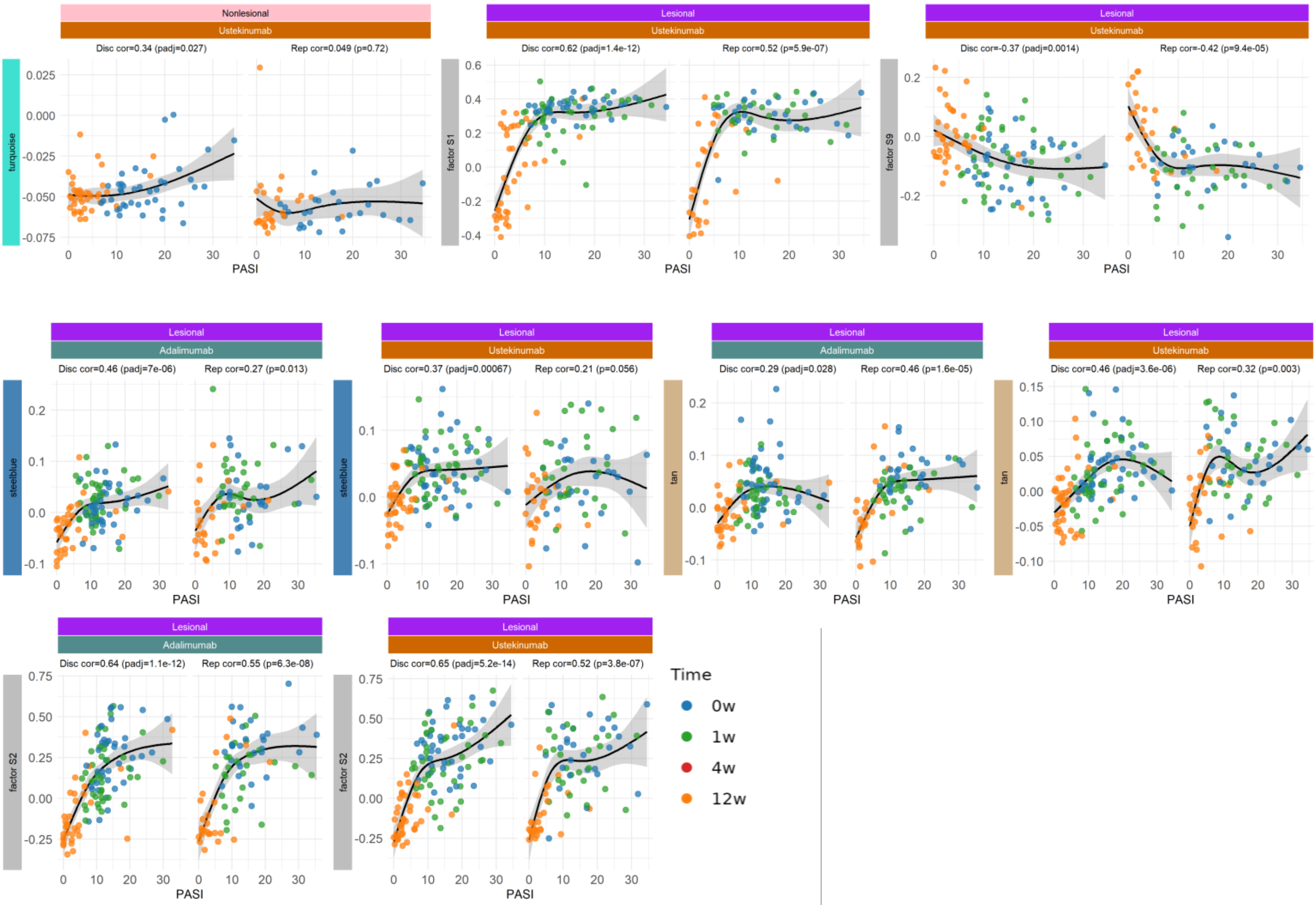

Supplementary Figure 9. Further exemplar module and latent factor-trait correlation plots.
Scatter plots of further exemplar WGCNA module and ICA factor trait associations with disease severity (PASI). Each point represents a single sample, is marked with a symbol according to drug cohort and colour-coded according to timepoint (blue = 0w, green = 1w, red = 4w, orange = 12w). The grey shading indicates 95% confidence intervals. The title banner provides information on the tissue and drug cohort. Module membership is denoted opposite the y axis. Curves represent natural-spline fits (3 d.f.) with 95 % confidence bands. *Abbreviations:* PASI, Psoriasis Area and Severity Index; WGCNA, Weighted gene correlation network analysis; ICA independent component analysis; w, weeks

a

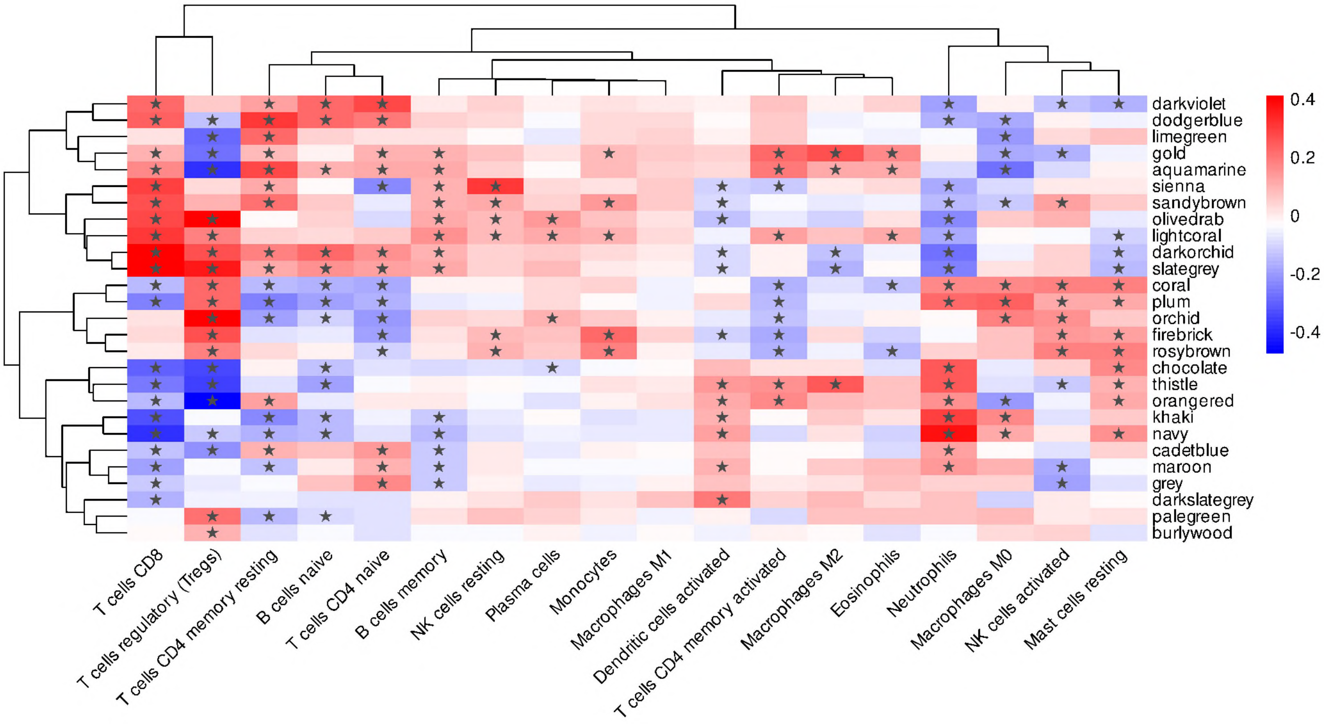

darkviolet
dodgerblue
limegreen
gold
aquamarine
sienna
sandybrown
olivedrab
lightcoral
darkorchid
slategrey
coral
plum
orchid
firebrick
rosybrown
chocolate
thistle
orangered
khaki
navy
cadetblue
maroon
grey
darkslategrey
palegreen
burlywood
0.4
0.2
0
-0.2
-0.4
T cells CD8
T cells regulatory (Tregs)
T cells CD4 memory resting
B cells naive
T cells CD4 naive
B cells memory
NK cells resting
Plasma cells
Monocytes
Macrophages M1
Dendritic cells activated
T cells CD4 memory activated
Macrophages M2
Eosinophils
Neutrophils
Macrophages M0
NK cells activated
Mast cells resting

b

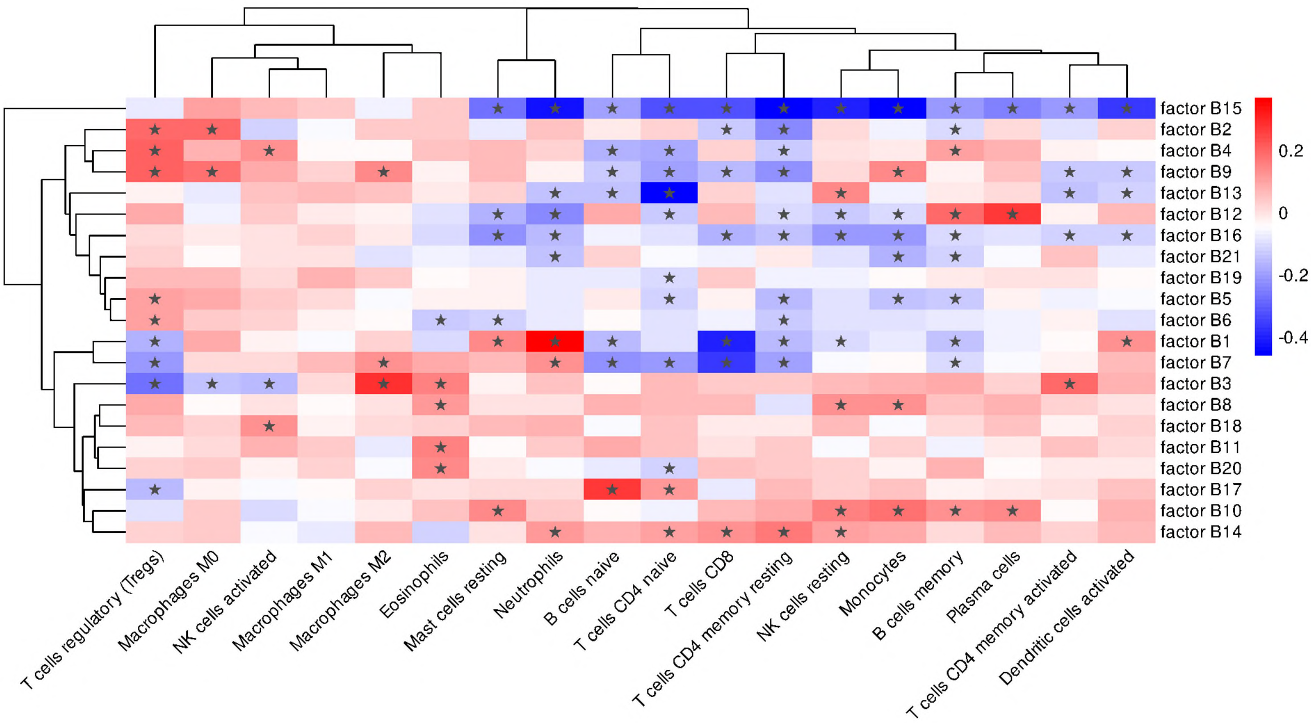

factor B15
factor B2
factor B4
factor B9
factor B13
factor B12
factor B16
factor B21
factor B19
factor B5
factor B6
factor B1
factor B7
factor B3
factor B8
factor B18
factor B11
factor B20
factor B17
factor B10
factor B14
0.2
0
-0.2
-0.4
T cells regulatory (Tregs)
Macrophages M0
NK cells activated
Macrophages M1
Macrophages M2
Eosinophils
Mast cells resting
Neutrophils
B cells naive
T cells CD4 naive
T cells CD8
T cells CD4 memory resting
NK cells resting
Monocytes
B cells memory
Plasma cells
T cells CD4 memory activated
Dendritic cells activated

Supplementary Figure 10. Cell type deconvolution of the gene modules and latent factors in blood. We derived predicted cell-type fractions for the blood RNA-Seq data using CibersortX and the LM22 reference dataset. We then correlated the predicted cell-type fractions with **(a)** module eigengenes, derived by WGCNA, and **(b)** latent factor values, derived by ICA. Asterisks (*) indicate statistical significance (adjusted p-value<0.05).
Abbreviations: WGCNA, weighted gene correlation network analysis; ICA, independent component analysis.

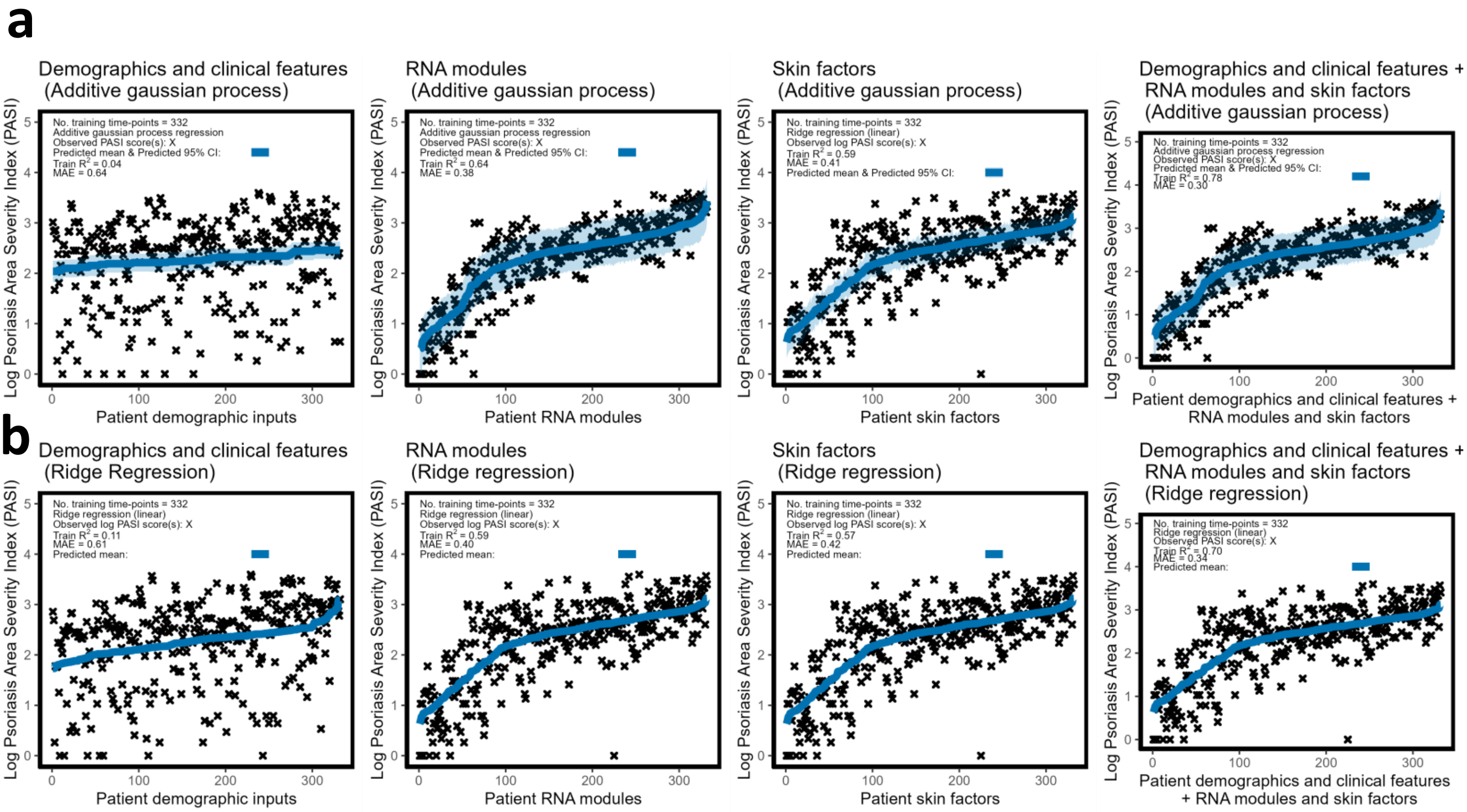

Supplementary Figure 11. **Prediction of PASI based on module eigengenes and latent factors in lesional skin and demographic and clinical features using additive gaussian process regression and linear ridge regression.** **(a)** Additive gaussian process regression models depicting the relationship between module eigengenes and latent factor values in lesional skin and demographic and clinical features (discovery and replication cohorts combined) and log scaled PASI. Top row: the first regression model was trained on patient demographic and clinical information. The next two regression models were trained on module eigengenes or latent factors. The third regression model was trained on all features. **(b)** Ridge regression (baseline) models depicting the relationship between patient inputs and PASI. The observed log PASI scores from the training dataset (n=332 time-points) are depicted by the cross symbols. The shaded regions represent the additive gaussian process models predicted 95% confidence intervals.

Abbreviations: PASI, psoriasis area and severity index.

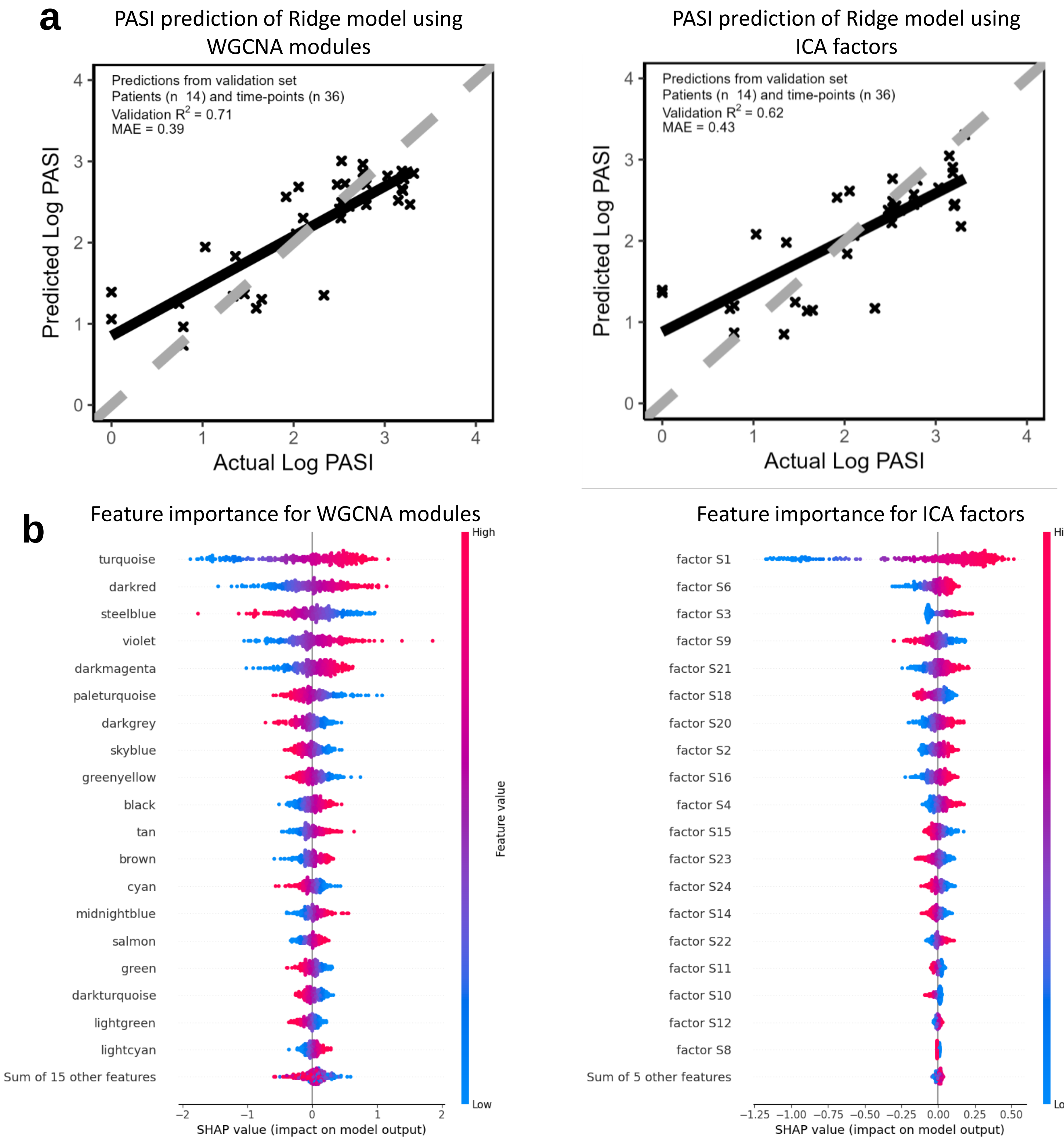

Supplementary Figure 12. Prediction of PASI based on module eigengenes and latent factors in lesional skin and demographic and clinical features using linear ridge regression.

(a) Linear (ridge) regression models depicting the relationship between module eigengenes and latent factor values in lesional skin and demographic and clinical features (discovery and replication cohorts combined) and log scaled PASI. The performance comparison between the predicted and actual log PASI scores were optimized on the validation dataset. Among all features, modules eigengenes and latent factors gave the best performance. Demographic and clinical features made no difference to performance (Supplementary Fig. 12). The observed log PASI scores from the training dataset (n=332 time-points) are depicted by the cross symbols. **(b)** Scatter plots show the relative feature importance determined using the SHAP (SHapley Additive exPlanations) method. SHAP values were calculated using a model-agnostic approach, where model features are independently altered and the resulting change in the predicted output is recorded while keeping other features unchanged. SHAP values on the right side of the x-axis indicate features decreasing PASI, whilst those shifting right indicate the opposite.
Abbreviations: PASI, psoriasis area and severity index; SHAP, SHapley Additive exPlanations.

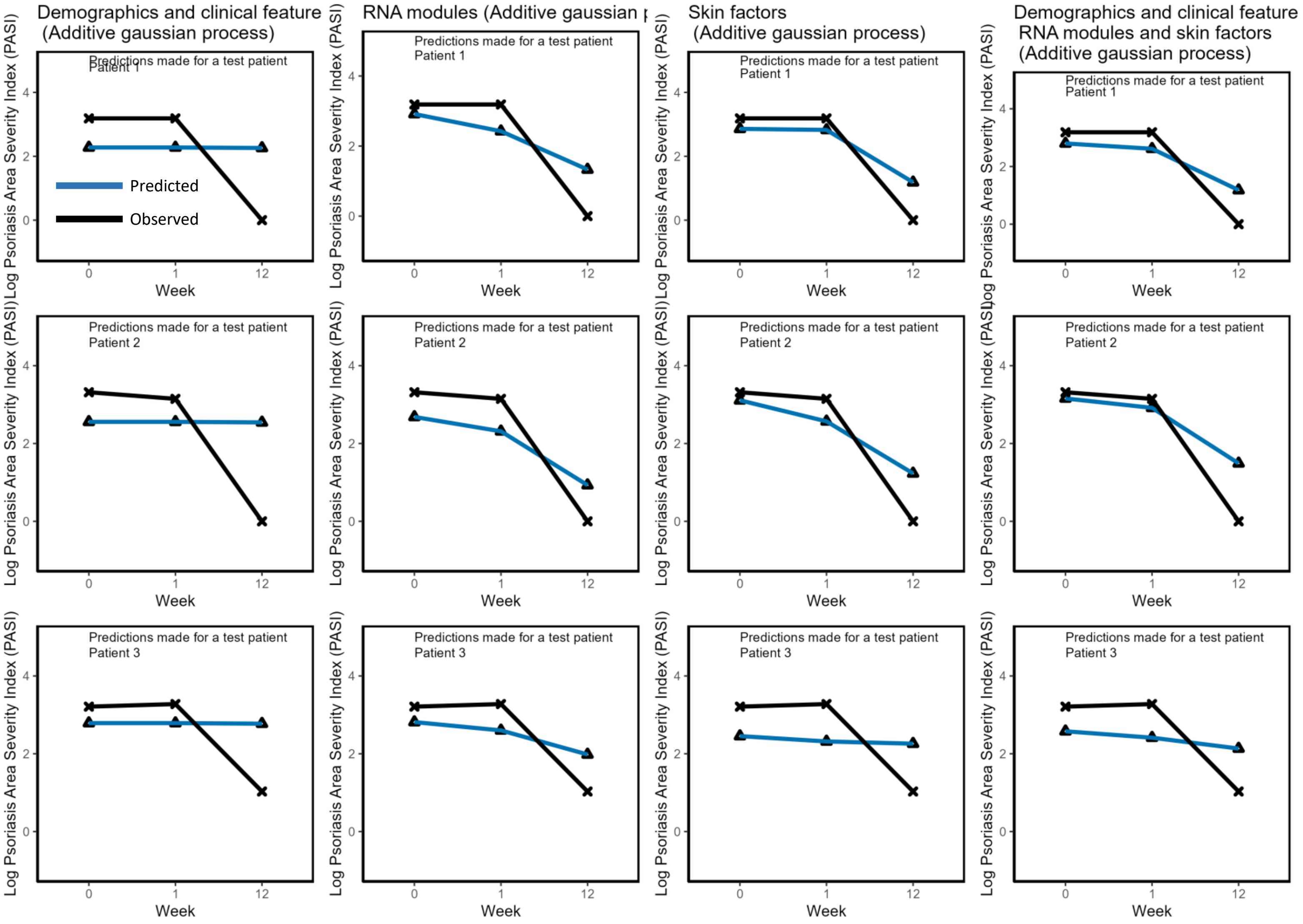

Supplementary Figure 13. Predicted PASI trajectories across time based on gaussian process regression. Patient trajectories predicted by the additive gaussian process regression models from a randomly shuffled 10-fold held-out testing dataset. The first regression model was trained on patient demographic and clinical information. The next two regression models were trained on module eigengenes or latent factors. The final regression model was trained on all features.

Abbreviations: PASI, psoriasis area and severity index.

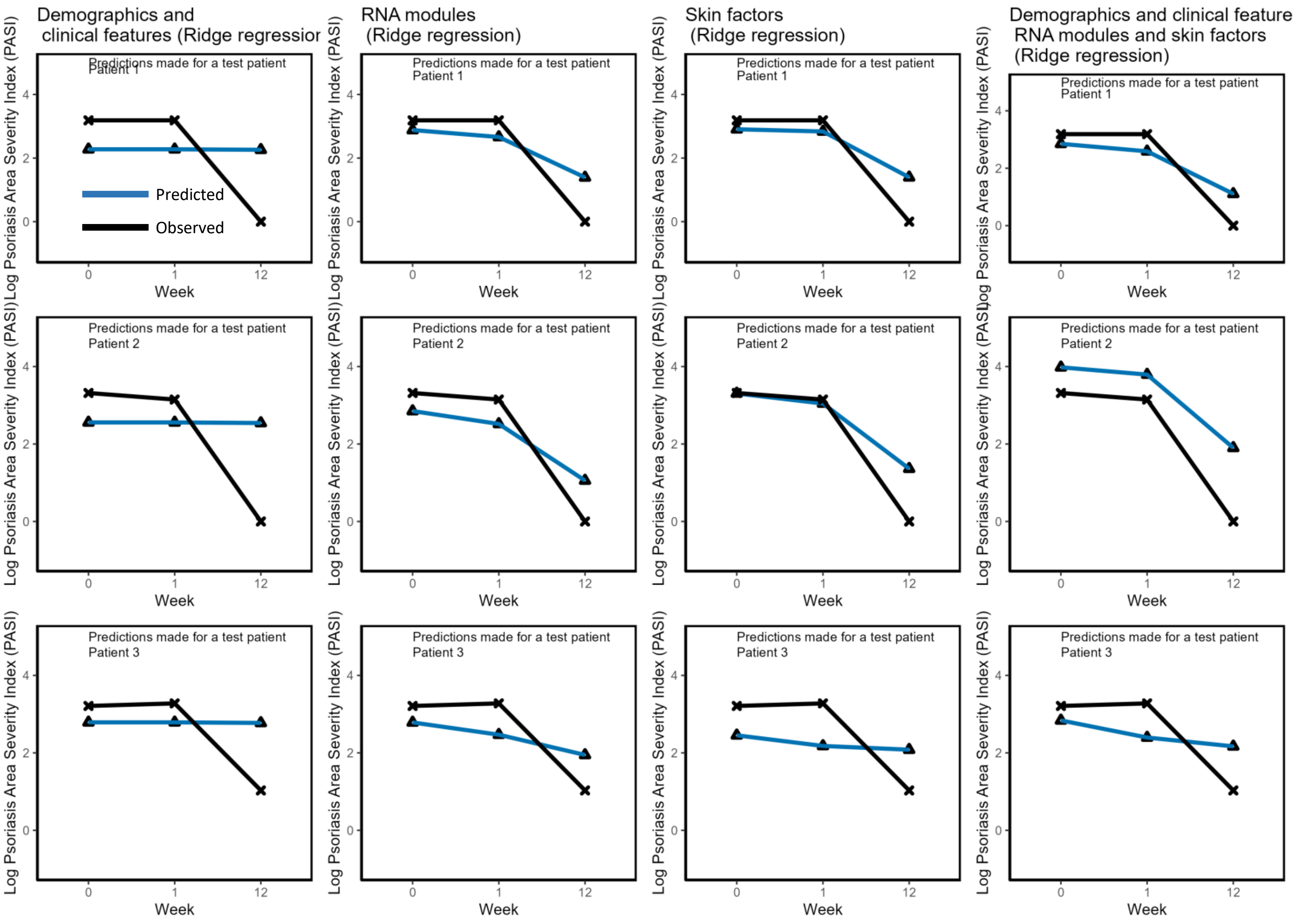

Supplementary Figure 14. Predicted PASI trajectories across time based on linear ridge regression. Patient trajectories predicted by the linear ridge regression models from a randomly shuffled 10-fold held-out testing dataset. The first regression model was trained on patient demographic and clinical information. The next two regression models were trained on module eigengenes or latent factors. The final regression model was trained on all features.

Abbreviations: PASI, psoriasis area and severity index.

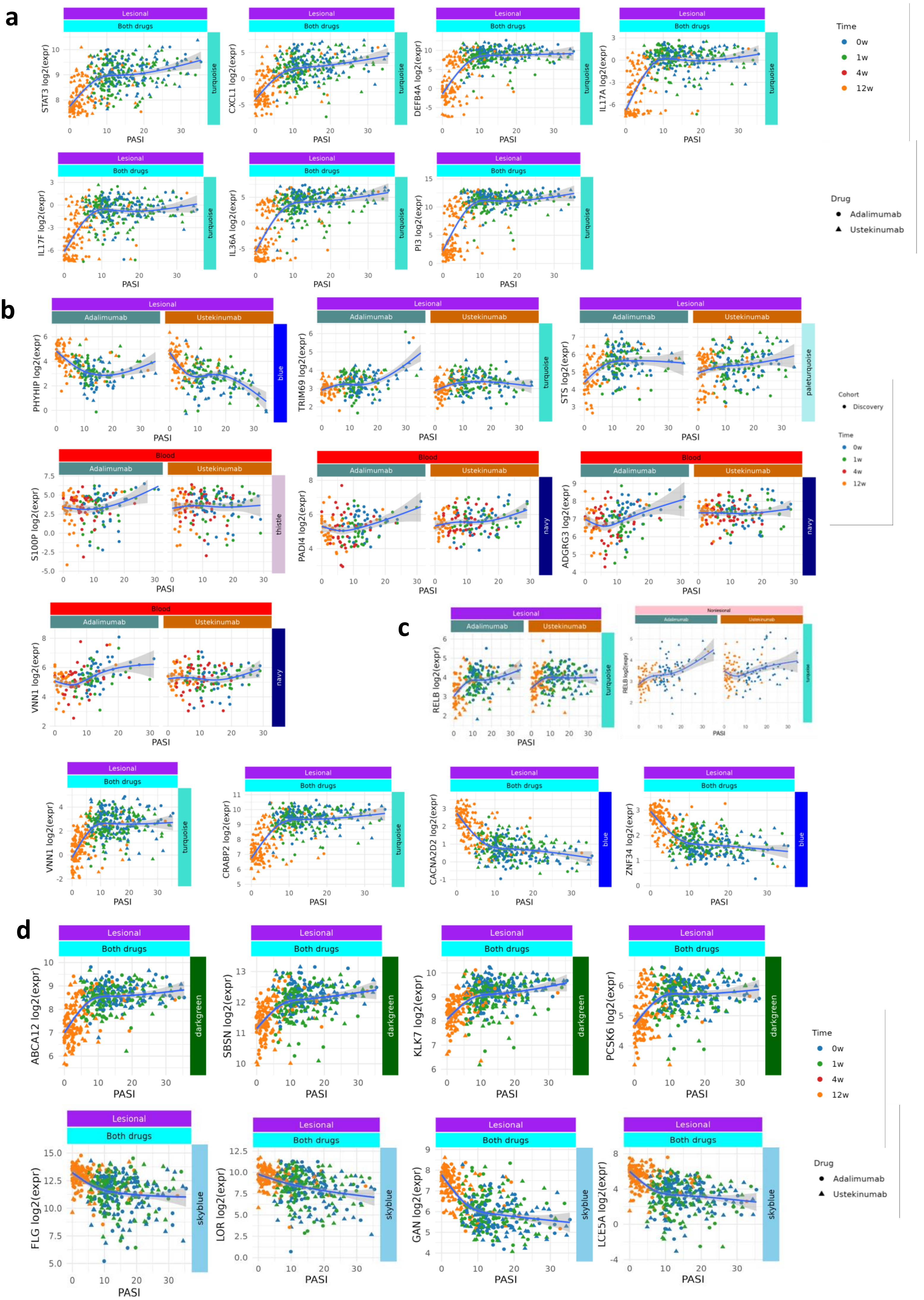

a
Lesional
Both drugs
STAT3 log2(expr)
CXCL1 log2(expr)
DEFB4A log2(expr)
IL17A log2(expr)
IL17F log2(expr)
IL36A log2(expr)
PI3 log2(expr)
PASI
turquoise
Time
0w
1w
4w
12w
Drug
Adalimumab
Ustekinumab
b
Adalimumab
Ustekinumab
PHYHIP log2(expr)
blue
TRIM69 log2(expr)
STS log2(expr)
paleturquoise
Cohort
Discovery
Blood
S100P log2(expr)
thistle
PADI4 log2(expr)
navy
ADGRG3 log2(expr)
VNN1 log2(expr)
c
RELB log2(expr)
Nonlesional
VNN1 log2(expr)
CRABP2 log2(expr)
CACNA2D2 log2(expr)
ZNF34 log2(expr)
d
ABCA12 log2(expr)
SBSN log2(expr)
KLK7 log2(expr)
PCSK6 log2(expr)
darkgreen
FLG log2(expr)
LOR log2(expr)
GAN log2(expr)
LCE5A log2(expr)
skyblue

Supplementary Figure 15. Further examples of gene-level associations with disease severity in skin and blood.

Scatter plots of the log2-CPM expression against PASI of further example disease severity-associated genes. Each point represents a single sample, is marked with a symbol according to drug cohort and colour-coded according to timepoint (blue = 0w, green = 1w, red = 4w, orange = 12w). The grey shading indicates 95% confidence intervals. The title banner provides information on the tissue and drug cohort. Module membership is denoted opposite the y axis. **(a)** Exemplars; **(b)** drug-specific examples; **(c)** PASI-associated signature genes, identified through SHAP analysis of blue and turquoise modules (Fig. 4D); **(d)** skin barrier genes that were also members of the darkgreen or skyblue modules in skin.

Curves represent natural-spline fits (3 d.f.) with 95 % confidence bands.

Abbreviations: CPM, counts per million; PASI, psoriasis area and severity index; w, week; SHAP, SHapley Additive exPlanations.

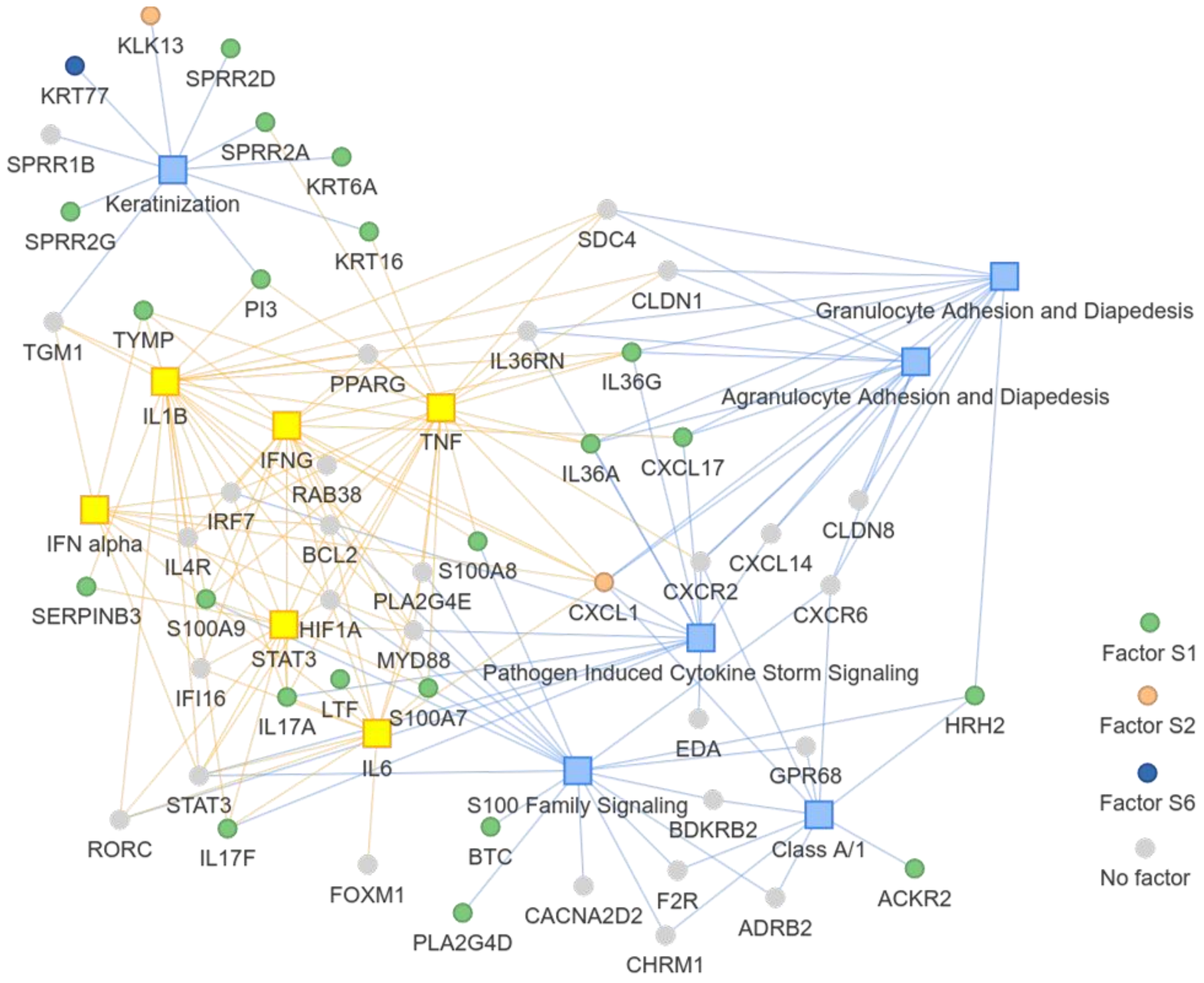

Supplementary Figure 16. Network analysis of the top disease severity-associated genes in lesional skin for the combined drug cohort.

Genes (circles) are coloured according to associated skin factors, upstream regulators (yellow squares) and canonical pathways (blue squares) were identified by IPA from all strongly disease severity-associated genes.

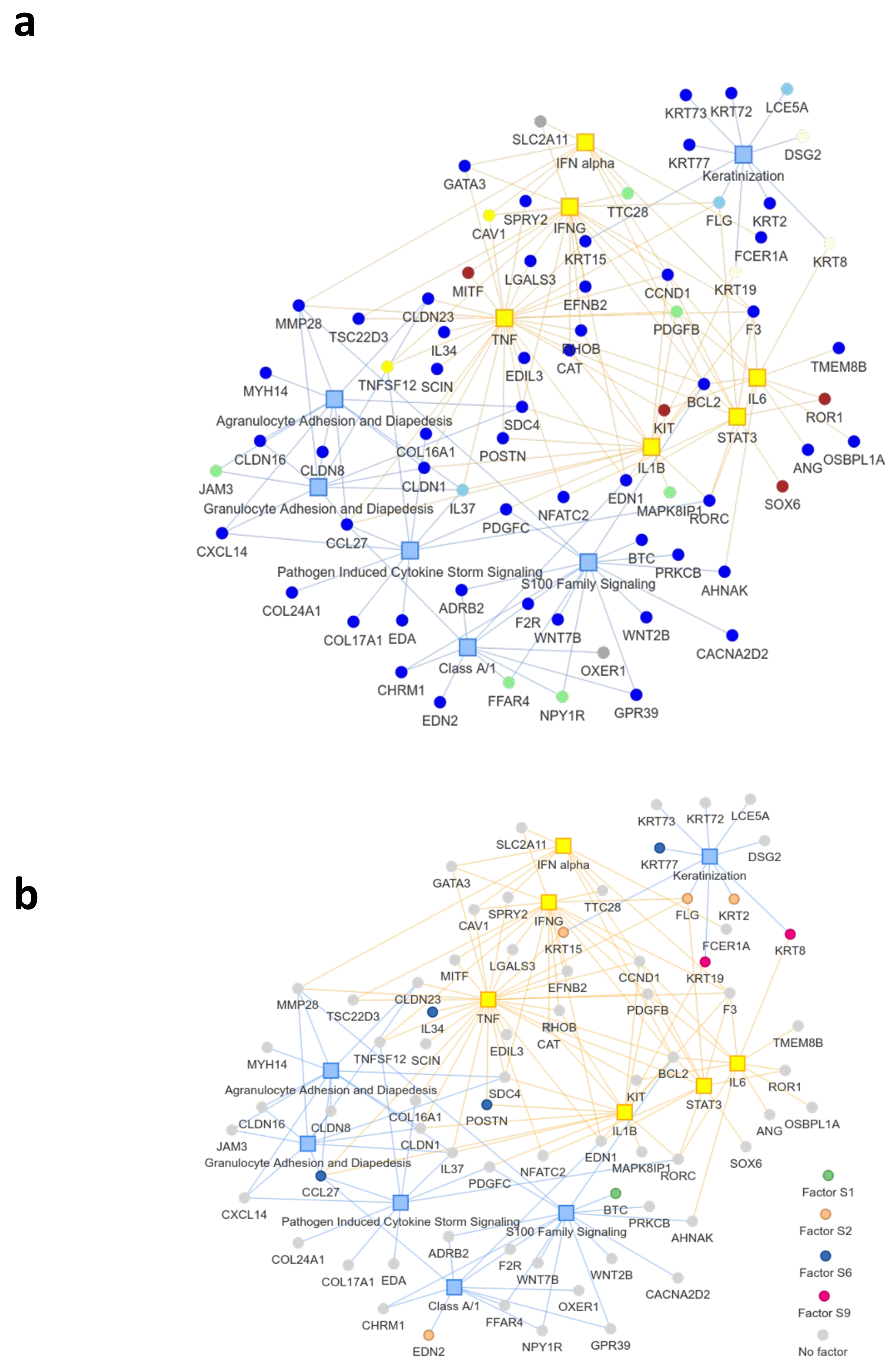

Supplementary Figure 17. Network analysis of top negative disease severity-associated genes in lesional skin for the combined drug cohort.

Networks are colour coded **(a)** according their WGCNA module, and **(b)** according to associated skin factors. Genes (circles) are coloured according to associated skin factors, upstream regulators (yellow squares) and canonical pathways (blue squares) were identified by IPA from all strongly disease severity-associated genes.

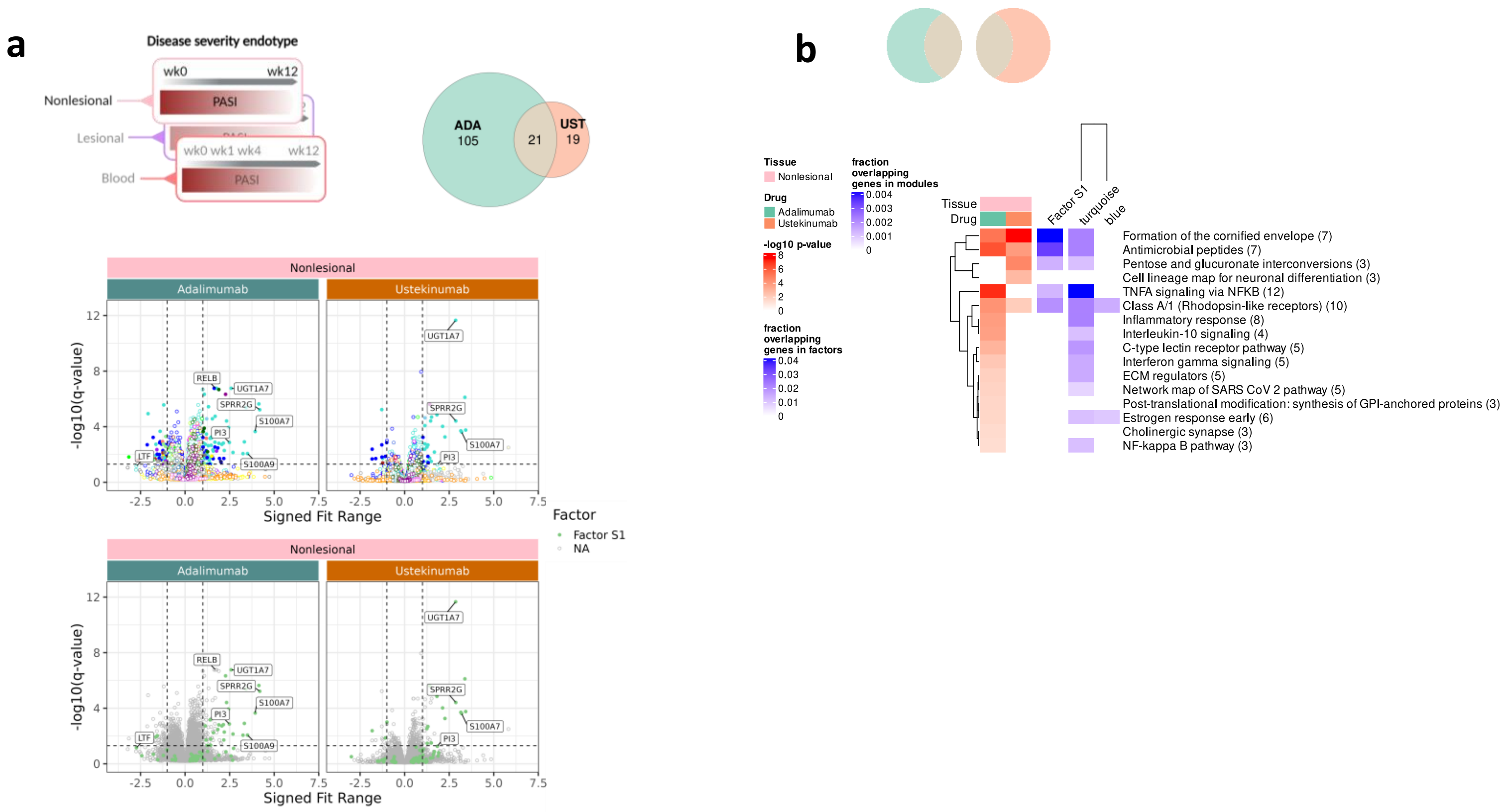

Supplementary figure 18. Disease severity endotypes in non-lesional skin (NLS).
(a) (Upper left) Schematic. (Upper right) Venn Diagram showing overlap of genes in NLS with significant (q-value<0.05) non-linear association (natural spline with 3 degrees of freedom) of expression with disease severity (DS), measured by PASI, in the adalimumab and ustekinumab cohorts. (Lower) Volcano plots of gene expression within the discovery and replication cohorts, facetted by drug cohort and plotted against signed fit range (overall direction and magnitude of DS association); points are coloured according to the WGCNA modules and ICA latent factors; example genes are labelled. **(b)** Metascape pathways from all co-expressed strongly DS-associated genes in each drug cohort, broken down by WGCNA module. The red heatmap shows the –log10 p-value, the blue shows the fraction of overlapping genes. The blue heatmap is normalised by the number of genes in the union of WGCNA module and Metascape pathway. *Abbreviations:* ADA, adalimumab; UST, ustekinumab; NLS, non-lesional skin; DS, disease severity; WGCNA, weighted gene correlation network analysis; ICA, independent component analysis.

a

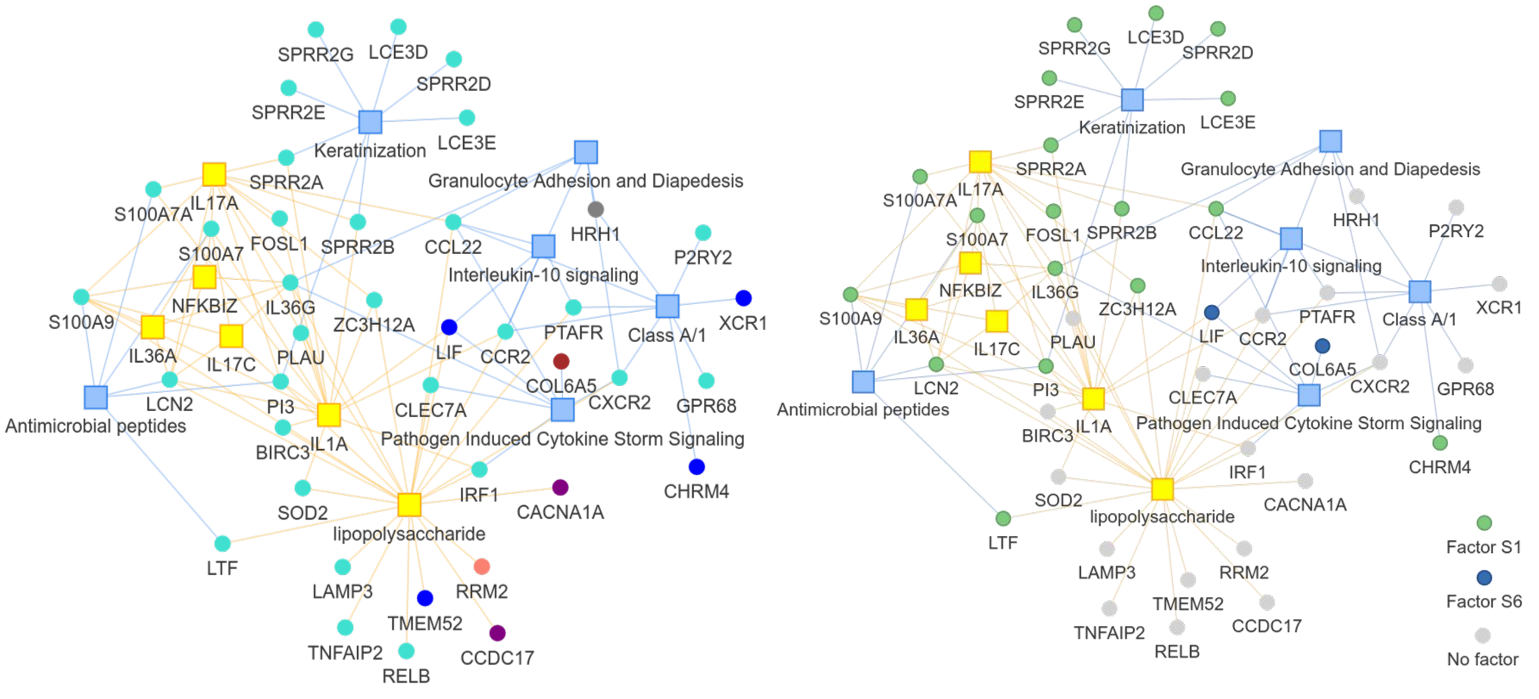

b

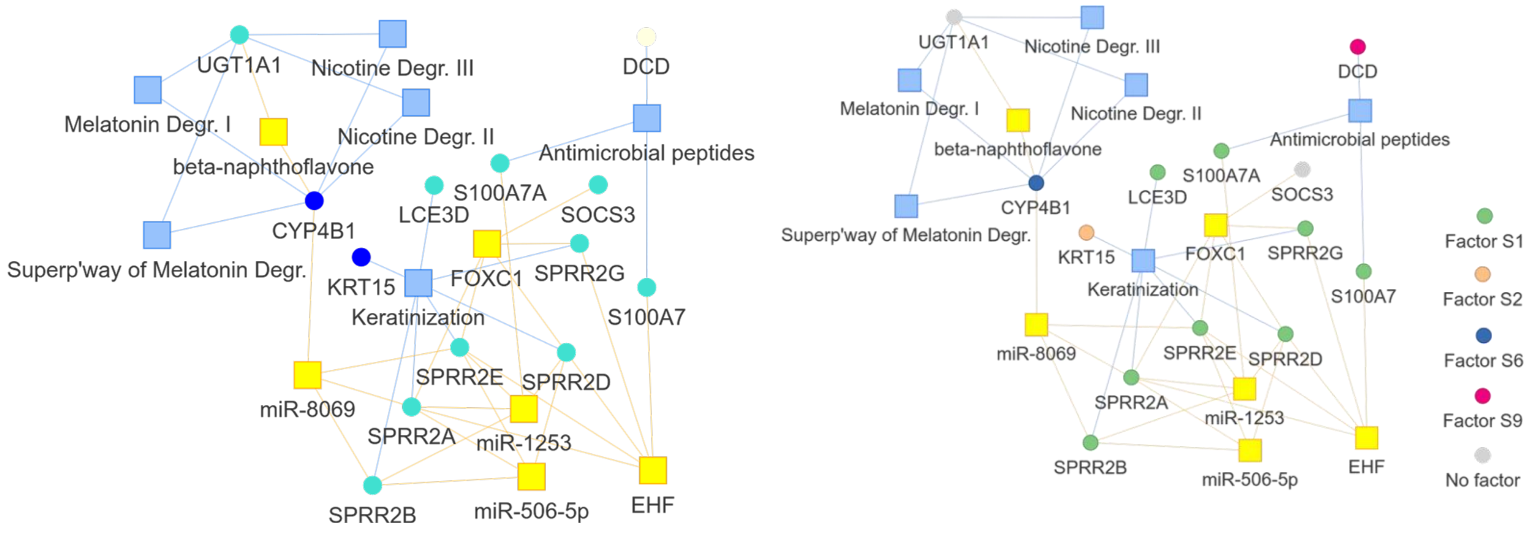

Supplementary Figure 19. Network analysis of top negative disease severity-associated genes in non-lesional skin (NLS).
Networks are shown for **(a)** the adalimumab cohort, colour coded according to their WGCNA module (left), and according to associated latent factors (right), and **(b)** the ustekinumab cohort, colour coded according to their WGCNA module (left), and according to associated skin factors (right). Significant genes (circles), upstream regulators (yellow squares) and canonical pathways (blue squares) were identified by IPA.
Abbreviations: WGCNA, weighted gene correlation network analysis; IPA, Ingenuity Pathway Analysis.

a

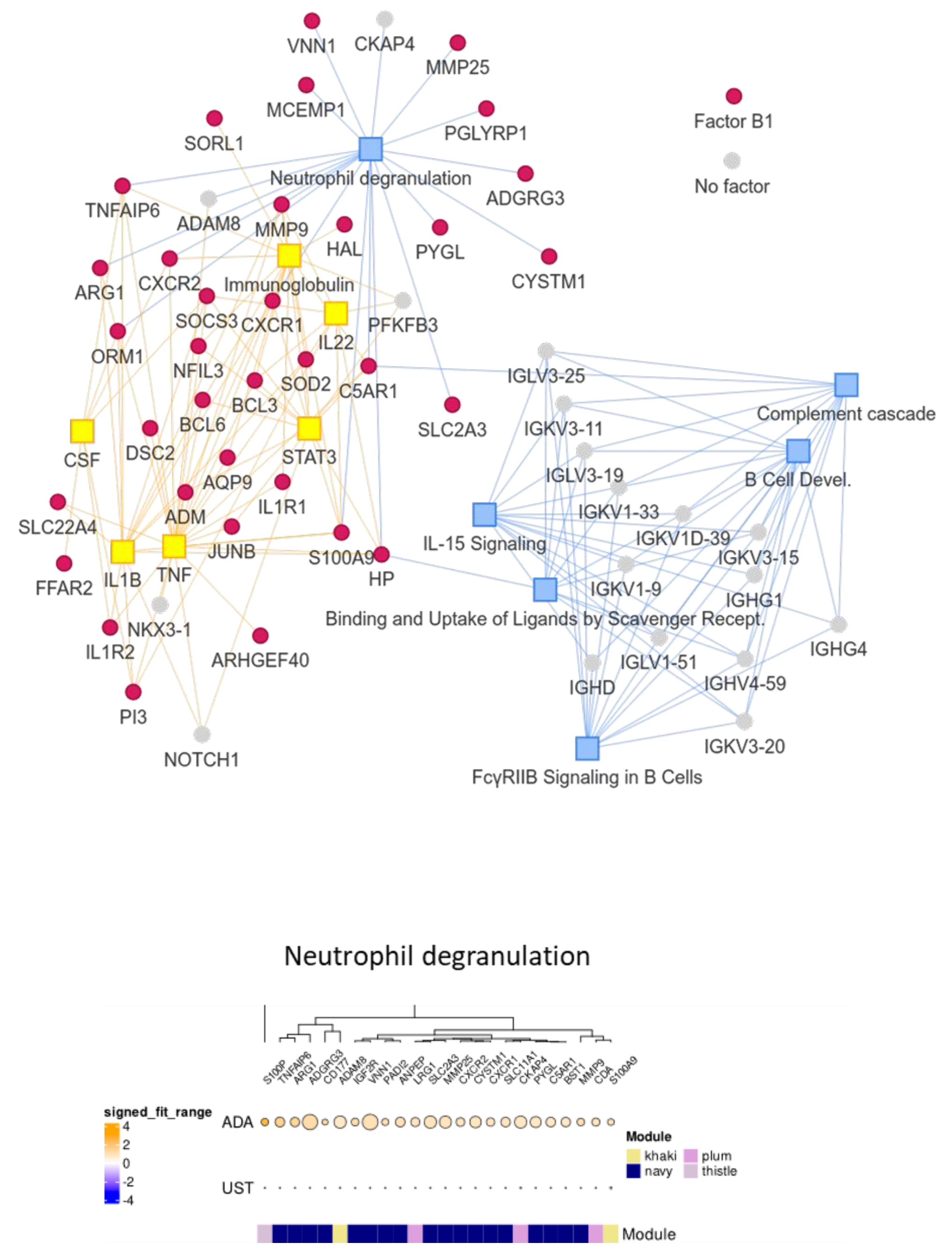

Supplementary Figure 20. Functional analysis of the top disease severity-associated genes in blood for the adalimumab cohort.

(a) Network analysis of the top disease severity-associated genes in blood for the adalimumab cohort. Genes (circles) are coloured according to associated latent factors, upstream regulators (yellow squares) and canonical pathways (blue squares) were identified by IPA from all strongly disease severity-associated genes. **(b)** Dot plots of the disease severity-associated genes within the Metascape neutrophil degranulation pathway. Gene expression is separated by drug cohort and clustered by signed fit range. The radius of the dot represents significance –log10 q-value and colour represents signed fit range. Module membership is recorded below each gene.

Abbreviations: IPA, Ingenuity Pathway Analysis.

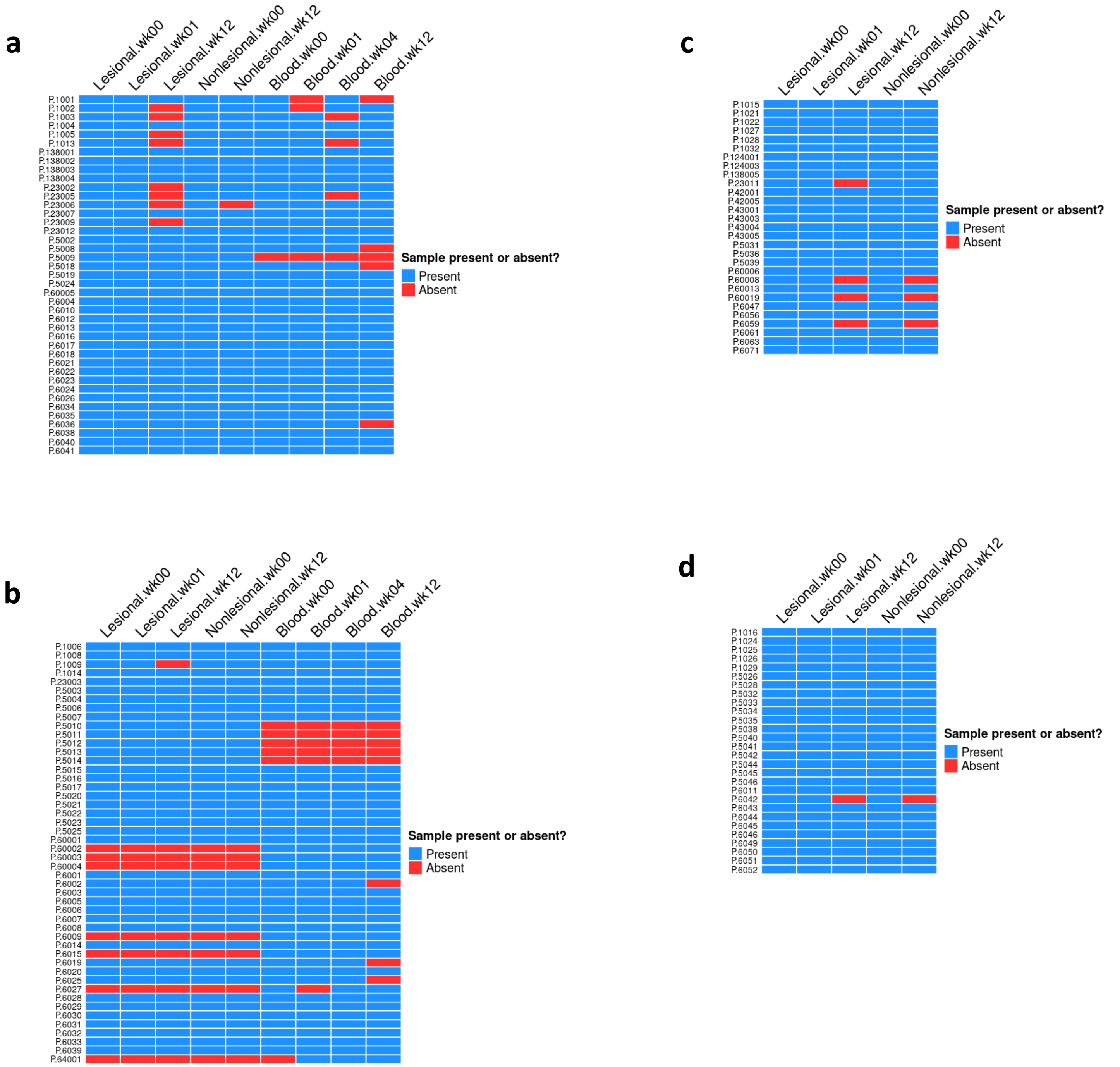

Supplementary figure 21. Graphical representations of RNA-Seq sample completeness. Graphs are shown for the **(a)** adalimumab group and **(b)** ustekinumab group in the discovery cohort, and the **(c)** adalimumab group and **(d)** ustekinumab group in the replication cohort. In each graph, patients are displayed on the x-axis and tissue-times are displayed on the y-axis. Blue indicates that a sample was collected, sequenced and used for analysis at the corresponding tissue-time for the corresponding patient; red indicates a missing sample for the corresponding tissue-time and patient.

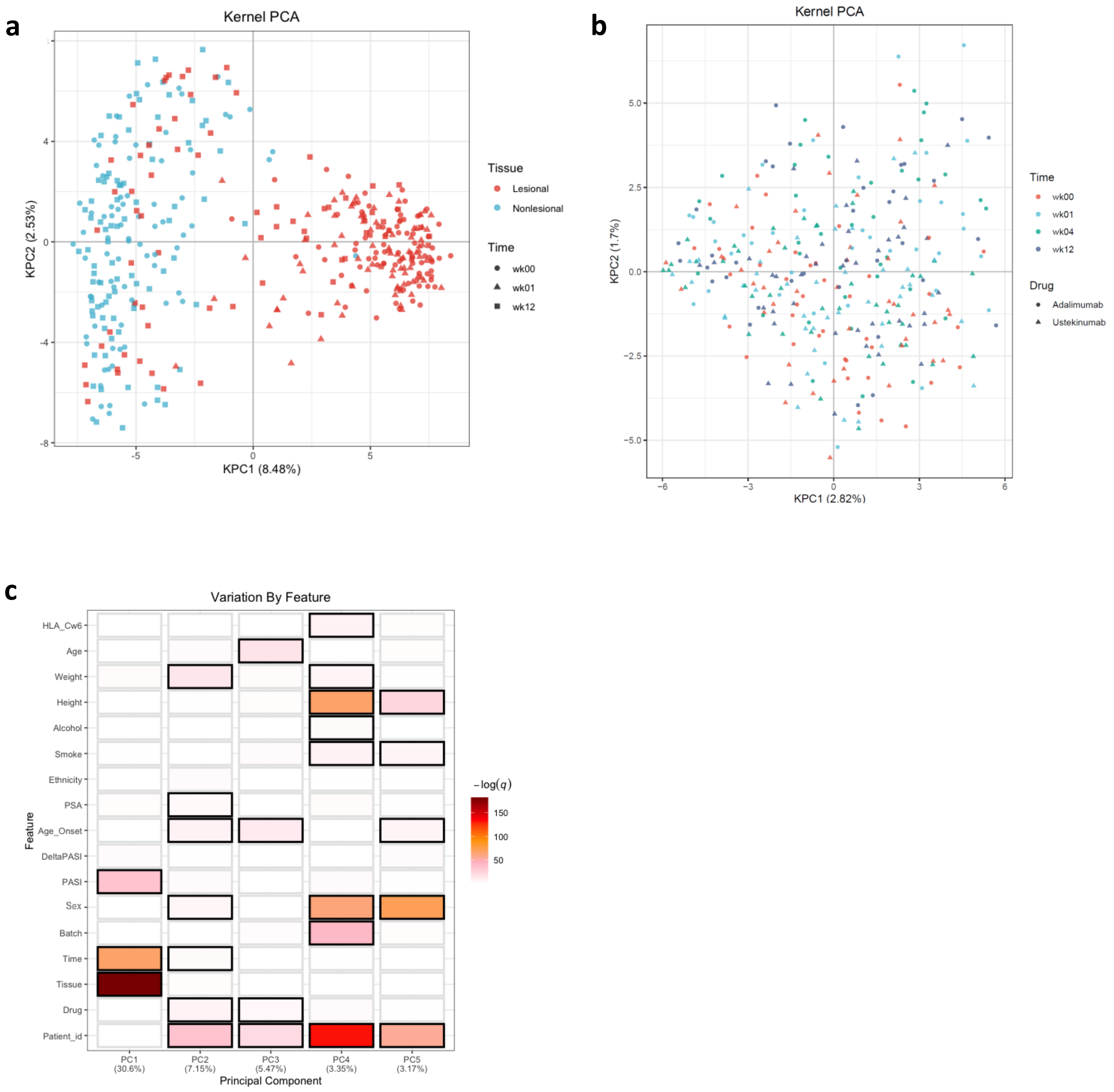

Supplementary Figure 22. Drivers of transcriptomic variation in skin and blood.
As an exploratory analysis, prior to conducting gene-level differential expression analysis or dimensionality reduction with WGCNA or ICA, we used Kernal PCA to explore the effect of tissue type, time point and drug on transcriptomic variation in the discovery cohort skin and blood RNA-Seq data. PCA plots of the first two principal components in **(a)** skin and **(b)** blood are shown. We used statistical analysis to investigate associations between the first five principal components in skin and an extended set of clinical and demographic traits; this is visualised in **(c)**.
Abbreviations: WGCNA, weighted gene correlation network analysis; ICA, independent component analysis; PCA, principal component analysis.

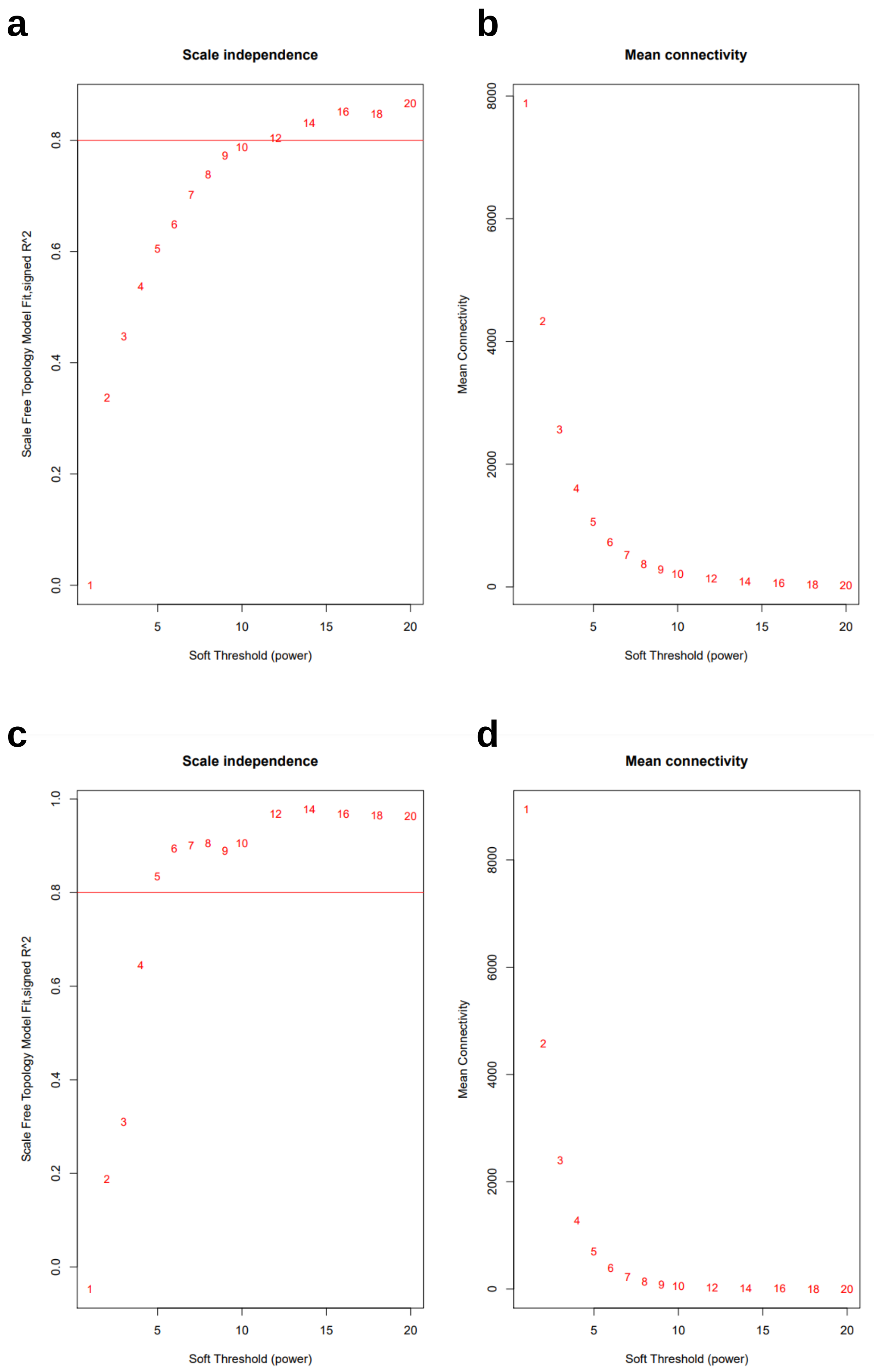

a
Scale independence
Scale Free Topology Model Fit,signed R^2
Soft Threshold (power)
b
Mean connectivity
Mean Connectivity
Soft Threshold (power)
c
Scale independence
Scale Free Topology Model Fit,signed R^2
Soft Threshold (power)
d
Mean connectivity
Mean Connectivity
Soft Threshold (power)

Supplementary Figure 23. Identifying appropriate soft-thresholding powers for WGCNA in skin and blood.

WGCNA involves creation of a gene-gene correlation matrix; prior to hierarchical clustering of this matrix, the correlation estimates are raised to a power in order to amplify the differences between high and low correlations; this power is chosen based on scale independence and mean connectivity. The scale independence **(a, c)** and mean connectivity **(b, d)** of the candidate soft-thresholding powers in skin **(a, b)** and blood **(c, d)**. The smallest power that passed an R2 threshold of 0.8 was selected for the downstream module identification steps. In skin this was 12 and in blood this was 5. Further details are available in the supplementary methods.

Supplementary Table 1a. Demographic information of study recruits for PSORT-D

		Overall n=89	Adalimumab n=41	Ustekinumab n=48
Biologic Naive, n (%)	No	27 (30.3)	10 (24.4)	17 (35.4)
	Yes	62 (69.7)	31 (75.6)	31 (64.6)
Ethnicity, n (%)	Asian or Asian British	6 (6.7)	3 (7.3)	3 (6.2)
	Black or Black British	2 (2.2)	1 (2.4)	1 (2.1)
	White	80 (89.9)	37 (90.2)	43 (89.6)
	Other	1 (1.1)		1 (2.1)
Sex, n (%)	F	33 (37.1)	15 (36.6)	18 (37.5)
	M	56 (62.9)	26 (63.4)	30 (62.5)
PsA, n (%)	Negative	62 (69.7)	27 (65.9)	35 (72.9)
	Positive	27 (30.3)	14 (34.1)	13 (27.1)
Flexural, n (%)	No	53 (59.6)	23 (56.1)	30 (62.5)
	Not Done	4 (4.5)	2 (4.9)	2 (4.2)
	Yes	32 (36.0)	16 (39.0)	16 (33.3)
Scalp, n (%)	No	17 (19.1)	9 (22.0)	8 (16.7)
	Not Done	4 (4.5)	2 (4.9)	2 (4.2)
	Yes	68 (76.4)	30 (73.2)	38 (79.2)
Palms, n (%)	No	66 (74.2)	33 (80.5)	33 (68.8)
	Not Done	4 (4.5)	2 (4.9)	2 (4.2)
	Yes	19 (21.3)	6 (14.6)	13 (27.1)
Soles, n (%)	No	67 (75.3)	32 (78.0)	35 (72.9)
	Not Done	4 (4.5)	2 (4.9)	2 (4.2)
	Yes	18 (20.2)	7 (17.1)	11 (22.9)
Nails, n (%)	No	25 (28.1)	13 (31.7)	12 (25.0)
	Not Done	4 (4.5)	2 (4.9)	2 (4.2)
	Yes	60 (67.4)	26 (63.4)	34 (70.8)
Age, mean (SD)		44.4 (12.3)	41.4 (10.3)	46.9 (13.4)
Age of onset, mean (SD)		22.6 (13.2)	21.2 (10.8)	23.9 (15.1)
wk00 PASI, mean (SD)		15.3 (5.9)	14.5 (5.2)	15.9 (6.4)
wk01 PASI, mean (SD)		13.7 (6.0)	12.4 (5.0)	14.8 (6.6)
wk04 PASI, mean (SD)		9.1 (5.7)	8.3 (5.2)	9.8 (6.0)
wk12 PASI, mean (SD)		3.9 (4.9)	4.1 (6.1)	3.8 (3.8)
wk00 DLQI, mean (SD)		17.5 (7.0)	16.9 (6.9)	17.9 (7.1)
wk01 DLQI, mean (SD)		15.3 (7.2)	14.3 (6.5)	16.2 (7.7)
wk04 DLQI, mean (SD)		9.7 (6.7)	7.9 (5.6)	11.2 (7.2)
wk12 DLQI, mean (SD)		4.2 (5.2)	3.6 (4.5)	4.6 (5.7)
Cw6, n (%)	Negative	46 (56.1)	23 (56.1)	23 (56.1)
	Positive	36 (43.9)	18 (43.9)	18 (43.9)
DeltaPASI, mean (SD)		0.7 (0.2)	0.8 (0.2)	0.7 (0.2)
Smoking, n (%)	No	27 (30.3)	12 (29.3)	15 (31.2)
	Yes	62 (69.7)	29 (70.7)	33 (68.8)
Drink Alcohol, n (%)	No	24 (27.0)	4 (9.8)	20 (41.7)
	Yes	65 (73.0)	37 (90.2)	28 (58.3)
Alcohol (units/week), mean (SD)		8.3 (16.7)	8.5 (10.2)	8.1 (20.8)
BMI, mean (SD)		30.8 (6.4)	31.6 (7.0)	30.1 (5.7)
BMI status, n (%)	Healthy weight (>=18.5 <30)	17 (19.3)	8 (19.5)	9 (19.1)
	Obese (>=30)	44 (50.0)	23 (56.1)	21 (44.7)
	Overweight (>=25 <30)	27 (30.7)	10 (24.4)	17 (36.2)

Supplementary Table 1b. Demographic information of study recruits for PSORT-R

		Overall n=57	Adalimumab n=29	Ustekinumab n=28
Biologic Naive, n (%)	No	8 (14.0)	1 (3.4)	7 (25.0)
	Yes	49 (86.0)	28 (96.6)	21 (75.0)
Ethnicity, n (%)	Asian or Asian British	2 (3.5)	1 (3.4)	1 (3.6)
	Other	2 (3.5)	2 (6.9)	
	White	52 (91.2)	26 (89.7)	26 (92.9)
	Black or Black British	1 (1.8)		1 (3.6)
Sex, n (%)	F	28 (49.1)	13 (44.8)	15 (53.6)
	M	29 (50.9)	16 (55.2)	13 (46.4)
PsA, n (%)	Negative	39 (68.4)	16 (55.2)	23 (82.1)
	Positive	18 (31.6)	13 (44.8)	5 (17.9)
Flexural, n (%)	No	24 (42.1)	14 (48.3)	10 (35.7)
	Yes	33 (57.9)	15 (51.7)	18 (64.3)
Scalp, n (%)	No	7 (12.3)	5 (17.2)	2 (7.1)
	Yes	50 (87.7)	24 (82.8)	26 (92.9)
Palms, n (%)	No	42 (73.7)	20 (69.0)	22 (78.6)
	Yes	15 (26.3)	9 (31.0)	6 (21.4)
Soles, n (%)	No	43 (75.4)	20 (69.0)	23 (82.1)
	Not Done	1 (1.8)	1 (3.4)	
	Yes	13 (22.8)	8 (27.6)	5 (17.9)
Nails, n (%)	No	24 (42.1)	11 (37.9)	13 (46.4)
	Yes	33 (57.9)	18 (62.1)	15 (53.6)
Age, mean (SD)		46.2 (12.5)	44.3 (11.6)	48.2 (13.4)
Age of onset, mean (SD)		24.1 (15.4)	22.0 (13.5)	26.1 (17.1)
wk00 PASI, mean (SD)		16.2 (7.6)	16.2 (7.2)	16.3 (8.2)
wk01 PASI, mean (SD)		14.4 (6.9)	14.0 (6.8)	14.7 (7.1)
wk04 PASI, mean (SD)		9.1 (5.1)	8.6 (4.7)	9.6 (5.5)
wk12 PASI, mean (SD)		3.9 (4.4)	4.2 (4.8)	3.7 (4.0)
wk00 DLQI, mean (SD)		17.0 (6.1)	17.9 (5.4)	16.1 (6.8)
wk01 DLQI, mean (SD)		14.3 (6.7)	14.1 (6.4)	14.5 (7.1)
wk04 DLQI, mean (SD)		8.8 (6.7)	8.0 (5.4)	9.6 (7.8)
wk12 DLQI, mean (SD)		3.4 (4.2)	4.6 (5.2)	2.1 (2.1)
Cw6, n (%)	Negative	27 (48.2)	12 (41.4)	15 (55.6)
	Positive	29 (51.8)	17 (58.6)	12 (44.4)
DeltaPASI, mean (SD)		0.7 (0.3)	0.7 (0.3)	0.7 (0.3)
Smoking, n (%)	No	21 (36.8)	12 (41.4)	9 (32.1)
	Yes	36 (63.2)	17 (58.6)	19 (67.9)
Drink Alcohol, n (%)	No	21 (36.8)	16 (55.2)	5 (17.9)
	Yes	36 (63.2)	13 (44.8)	23 (82.1)
Alcohol (units/week), mean (SD)		7.1 (10.6)	4.3 (7.9)	10.1 (12.2)
BMI, mean (SD)		32.2 (8.5)	33.7 (8.7)	30.7 (8.2)
BMI status, n (%)	Healthy weight (>=18.5 <30)	13 (22.8)	4 (13.8)	9 (32.1)
	Obese (>=30)	29 (50.9)	17 (58.6)	12 (42.9)
	Overweight (>=25 <30)	15 (26.3)	8 (27.6)	7 (25.0)

Supplementary Table 2: PASI prediction performance of Ridge models. Shown are means ± SD for 20 randomly shuffled 10-fold testing datasets.

Inputs	Test MAE	Test R^2
Demographics/clinical features<br>WGCNA modules<br>ICA factors	0.47 ± 0.10	0.50 ± 0.10
All WGCNA modules	0.45 ± 0.06	0.54 ± 0.09
All ICA factors	0.46 ± 0.10	0.50 ± 0.11

Supplementary Table 3: Performance metrics for modules at the gene-level using additive Gaussian process models. Mean performance metrics ± SD for testing datasets.

Model inputs	Test MAE	Test R^2
Top 10 Factor S1 genes	0.47 ± 0.06	0.40 ± 0.10
Top 10 Factor S2 genes	0.48 ± 0.06	0.37 ± 0.15
Top 10 Factor S9 genes	0.54 ± 0.08	0.21 ± 0.16
Top 10 Factor S6 genes	0.51 ± 0.08	0.26 ± 0.14
Top 10 Factor S8 genes	0.62 ± 0.09	0.04 ± 0.09
Top 10 Factor S21 genes	0.64 ± 0.08	-0.02 ± 0.05

Supplementary Table 4: Performance metrics for modules at the gene-level using additive Gaussian process models. Mean performance metrics ± SD for testing datasets.

Model inputs	Test MAE	Test R^2
Top 10 Turquoise genes	0.43 ± 0.06	0.47 ± 0.13
Top 10 Violet genes	0.52 ± 0.07	0.29 ± 0.13
Top 10 Steelblue genes	0.57 ± 0.07	0.16 ± 0.14
Top 10 Blue genes	0.47 ± 0.07	0.44 ± 0.12
Top 10 Tan genes	0.50 ± 0.06	0.33 ± 0.09

Supplementary Table 5: Pearson correlations between factors and baseline PASI

Factor	Correlation	P-value	Adjusted p-value
**S18**	-0.278	0.00092	0.01286
**S2**	0.275	0.00107	0.01286
**S1**	0.245	0.00366	0.02925
**S17**	0.228	0.00685	0.04110
**S10**	-0.170	0.04587	0.22020
**S23**	-0.149	0.07913	0.31585
**S16**	0.143	0.09212	0.31585
**S21**	0.125	0.14280	0.42841
**S12**	0.109	0.20286	0.51030
**S13**	0.097	0.25614	0.51030
**S9**	-0.096	0.26254	0.51030
**S24**	-0.093	0.27603	0.51030
**S4**	-0.090	0.29293	0.51030
**S14**	-0.089	0.29768	0.51030
**S3**	-0.071	0.40563	0.63005
**S8**	-0.069	0.42003	0.63005
**S15**	0.060	0.48076	0.67871
**S20**	-0.056	0.51191	0.68255
**S19**	0.045	0.59873	0.74658
**S5**	0.042	0.62215	0.74658
**S11**	-0.031	0.71812	0.82071
**S6**	0.021	0.80188	0.87478
**S7**	0.011	0.90199	0.94121
**S22**	0.004	0.96012	0.96012

PSORT Consortium Membership

Executive committee

Jonathan Barker (St. Johns Institute of Dermatology, Kings College London, London, UK)
Michael Barnes (Centre for Translational Bioinformatics, William Harvey Research Institute, Queen Mary University of London, Charterhouse Square, London, UK)
Paola Di Meglio (St. Johns Institute of Dermatology, Kings College London, London, UK)
Richard Emsley (Institute of Psychiatry, Psychology & Neuroscience, Kings College London, London, UK)
Chris Griffiths (The Manchester Centre for Dermatology Research, The University of Manchester; Manchester, UK)
Nick Reynolds (Institute of Translational and Clinical Medicine, Faculty of Medical Sciences, Newcastle University, Newcastle upon Tyne, UK)
Catherine Smith (St. Johns Institute of Dermatology, Kings College London, London, UK)
Richard Warren (The Manchester Centre for Dermatology Research, The University of Manchester; Manchester, UK)

Recruiting Principal Investigators (not part of executive committee)

Dr Anna Chapman (Lewisham & Greenwich Trust)
Dr Rob Ellis (South Tees Hospitals NHS Foundation Trust)
Dr Abigail Fogo (Kingston Hospital NHS Foundation Trust)
Dr Bronwyn Hughes (Portsmouth Hospitals University NHS Trust)
Dr Evmorfia Ladoyanni (Dudley Group NHS Foundation Trust)
Dr Philip Laws (Leeds Teaching Hospitals NHS Trust)
Dr Richard Parslew (Liverpool University Hospitals NHS Foundation Trust)
Dr Gayathri Perera (Chelsea and Westminister Hospital NHS Foundation Trust)
