# Supplementary material for: Transcriptomic profiling and machine learning uncover gene signatures of psoriasis endotypes and disease severity
Source: Commun Med (Lond). 2026 Jan 21;6:65. doi: 10.1038/s43856-025-01325-4 (PMC12852803; doi:10.1038/s43856-025-01325-4)
Supplement: Supplementary file 3 — Description of supplementary data files [file 43856_2025_1325_MOESM3_ESM.docx]

**Description of Supplementary files**

Supplementary Data 1. WGCNA module data.xlsx

This file is an excel workbook with eight sheets: (i) the genes assigned to each skin module; (ii) the eigengenes for the skin modules in the discovery cohort; (iii) the eigengenes for the skin modules in the replication cohort; (iv) the correlation of genes with the skin module eigengenes in the discovery cohort; (v) the correlation of genes with skin module eigengenes in the replication cohort; (vi) the genes assigned to each blood module; (vii) the eigengenes for the blood modules; (viii) the correlation of genes with the blood module eigengenes. These data were utilised in the analysis presented in figures 2-7.

Supplementary Data 2. ICA factor data.xlsx

This file is an excel workbook with six sheets: (i) the skin ICA factor values for all samples; (ii) the blood ICA factor values for all samples; (iii) the skin factor gene loadings; (iv) the blood factor gene loadings; (v) the most aligned features for the skin ICA factors; (vi) the most aligned features for the blood ICA factors. These data were utilised in the analysis presented in figures 2-7.

Supplementary Data 3. Module and factor-trait correlations.xlsx

This file is an excel workbook with six sheets: (i) the details of the traits used for module and factor-trait correlation analysis in skin; (ii) results from module-trait correlation analysis in skin; (iii) results from factor-trait correlation analysis in skin; (iv) the details of the traits used for module and factor-trait correlation analysis in blood; (v) results from module-trait correlation analysis in blood; (vi) results from factor-trait correlation analysis in blood. These data were utilised in the analysis presented in figure 2.

Supplementary Data 4. Metascape results for module and factor genes.xlsx

Results from Metascape enrichment analysis of WGCNA module and ICA factor genes in skin and blood. This data was used to derive the module and factor descriptors displayed in figure 2a.

Supplementary Data 5. Module and factor metadata.xlsx

Metadata for modules and factors in skin and blood with significant clinical associations. Metadata for each module and factor include: descriptive name, size (for modules), top functional enrichments from Metascape, most aligned genes, and direction of PASI association.

Supplementary Data 6. PASI differential expression results.xlsx

Differential expression analysis results from the PASI model in lesional skin, non-lesional skin and blood for both drug cohorts. These data were utilised in the analysis presented in figure 3 and figures 6-7.

Supplementary Data 7. Expression data for example PASI DEGs.xlsx

The file contains the expression data and associated sample metadata for the example PASI-associated genes that are plotted in figure 3 and figure 6.

Supplementary Data 8. Metascape results for PASI DEGs.xlsx

Results from Metascape enrichment analysis of PASI-associated DEGs. This data was required for construction of figure 7d.

Supplementary Data 9. IPA results for PASI DEGs.xlsx

Results from IPA enrichment analysis of PASI-associated DEGs. This data was required for construction of figures 6e and 7e.

Supplementary Data 10. BMI differential expression results.xlsx

Differential expression analysis results from the BMI model. These data were utilised in the analysis presented in figure 3.

Supplementary Data 11. HLA endotype data.xlsx

Data from the HLA-based endotype analysis that was used to construct figure 5.

Supplementary Data 12. GPR - PASI prediction of Gaussian process model using skin modules.xlsx

PASI prediction data from Gaussian Process Regression with skin WGCNA modules. This data was utilised in the analysis presented in figure 4a.

Supplementary Data 13. GPR - PASI prediction of Gaussian process model using skin factors.xlsx

PASI prediction data from Gaussian Process Regression with skin ICA factors. This data was utilised in the analysis presented in figure 4a.

Supplementary Data 14. GPR - Feature importance for skin modules.xlsx

SHAP feature importance data for the Gaussian Process Regression with skin WGCNA modules. This data was utilised in the analysis presented in figure 4b.

Supplementary Data 15. GPR - Feature importance for skin factors.xlsx

SHAP feature importance data for the Gaussian Process Regression with skin ICA factors. This data was utilised in the analysis presented in figure 4b.

Supplementary Data 16. GPR - PASI prediction importance for turquoise and blue genes.xlsx

PASI prediction data from Gaussian Process Regression with genes from the turquoise and blue modules in skin. This data was utilised in the analysis presented in figure 4c.

Supplementary Data 17. GPR - Gene signature from turquoise and blue.xlsx

SHAP feature importance data for the Gaussian Process Regression with genes from the turquoise and blue modules in skin. This data was utilised in the analysis presented in figure 4d.
